# Bacterial and Fungal Infections Promote the Bone Erosion Progression in Acquired Cholesteatoma Revealed by Metagenomic Next-Generation Sequencing

**DOI:** 10.3389/fmicb.2021.761111

**Published:** 2021-11-05

**Authors:** Hua Jiang, Chengpeng Wu, Jingjie Xu, Qi Wang, Lei Shen, Xunyan Ou, Hongyan Liu, Xu Han, Jun Wang, Wenchao Ding, Lidan Hu, Xiangjun Chen

**Affiliations:** ^1^Department of Otolaryngology, The Second Affiliated Hospital, Zhejiang University School of Medicine, Hangzhou, China; ^2^Eye Center of the Second Affiliated Hospital, Institute of Translational Medicine, Zhejiang University School of Medicine, Hangzhou, China; ^3^Hangzhou Matridx Biotechnology Co., Ltd., Hangzhou, China; ^4^The Children’s Hospital, Zhejiang University School of Medicine, National Clinical Research Center for Child Health, Hangzhou, China

**Keywords:** acquired cholesteatoma, metagenomic next-generation sequencing (mNGS), microbes, infection, *Aspergillus*

## Abstract

An acquired cholesteatoma generally occurs as a consequence of otitis media and eustachian tube dysfunction. Patients with acquired cholesteatoma generally present with chronic otorrhea and progressive conductive hearing loss. There are many microbes reportedly associated with acquired cholesteatoma. However, conventional culture-based techniques show a typically low detection rate for various pathogenetic bacteria and fungi. Metagenomic next-generation sequencing (mNGS), an emerging powerful platform offering higher sensitivity and higher throughput for evaluating many samples at once, remains to be studied in acquired cholesteatoma. In this study, 16 consecutive patients from January 2020 to January 2021 at the Second Affiliated Hospital of Zhejiang University School of Medicine (SAHZU) were reviewed. We detected a total of 31 microbial species in patients, mNGS provided a higher detection rate compared to culture (100% vs. 31.25%, *p* = 0.000034). As the severity of the patient’s pathological condition worsens, the more complex types of microbes were identified. The most commonly detected microbial genus was *Aspergillus* (9/16, 56.25%), especially in patients suffering from severe bone erosion. In summary, mNGS improves the sensibility to identify pathogens of cholesteatoma patients, and *Aspergillus* infections increase bone destruction in acquired cholesteatoma.

## Introduction

An acquired cholesteatoma generally occurs as a consequence of otitis media and eustachian tube dysfunction. It is characterized by chronic infection and temporal bone deterioration (Parisier, 1989). Patients with acquired cholesteatoma generally present with chronic otorrhea and progressive conductive hearing loss. In addition to hearing loss, the damaging effects of cholesteatoma can lead to serious complications, such as labyrinthitis, facial paralysis, and brain abscess (Inagaki and Paparella, 2009). Surgery is currently regarded as the only clinical treatment option for cholesteatoma patients, which is directed at eradication of the entrapped keratinized epithelium and keratin from the middle ear and mastoid spaces. The primary goal of surgery is to create a disease-free, or risk-free ears. Nevertheless, medical management is indicated in preparation for a definitive surgical procedure, with no recommendations available to either prevent or cure the disease (Welkoborsky, 2011).

There are various microbes reportedly closely linked to acquired cholesteatoma, most commonly such as *Pseudomonas aeruginosa* and *Staphylococcus aureus* (Ricciardiello et al., 2009). Although the etiology of cholesteatoma remains unknown, previous studies have demonstrated that microbial activity may contribute to the pathogenesis and clinical behavior of cholesteatoma (Likus et al., 2016; Kalcioglu et al., 2018). However, to date, studies on the microbes associated with cholesteatoma have relied on traditional culture-based techniques for characterizing the microbiota. Since conventional culture-based techniques have a typically low detection rate due to the unculturability of many bacteria and fungi, more sensitive methods, such as 16S rRNA gene sequencing for bacteria identification have been developed to characterize microbiota (Liu et al., 2011; Kalcioglu et al., 2018; Weiss et al., 2019). The limitation of these studies based on 16S rRNA is obvious, the fungi, viruses and parasites were not considered. More recently, metagenomic next-generation sequencing (mNGS) based on the shotgun approach has been surging used for clinical diagnosis of infections (Mai et al., 2017; Wilson et al., 2017; Fan et al., 2018). Notably, mNGS offers higher sensitivity and high-throughput for evaluating many samples at once, and can provide more (and more reliable) information through sequence data than that is available through traditional culture-based analysis.

To our knowledge, no published study has yet described a mNGS-based evaluation of cholesteatoma-associated microbial communities. In this study, we apply this novel technique to investigate the diversity and composition of microbiota that accompany with cholesteatoma in order to provide insight into how these microbes contribute to the pathogenesis of cholesteatoma.

## Materials and Methods

### Ethics Statement

This study was reviewed and approved by the Institutional Review Board of SAHZU. Written informed consent was obtained from every patient and all procedures were conducted in accordance with the Declaration of Helsinki.

### Case Series

A total of 16 consecutive patients with cholesteatoma received surgery in the otolaryngology department of the Second Affiliated Hospital of Zhejiang University School of Medicine (SAHZU) from January 2020 to January 2021. The cholesteatoma tissue samples were obtained from patients during surgery and were collected in a sterile container. The specimens were sent for microbial bacterial and fungal smear and in our microbiological laboratory and mNGS sequencings and bioinformatic analysis by *Hangzhou Matridx Biotechnology Co*. All patients received pre-operative temporal bone CT scan and CT scores based on bone erosion (Table 1) were recorded.

**TABLE 1:** Scores of temporal CT scan.

Score	Pathological phenotype of CT scan	Estimated lesion area
1	Scutum and/or auditory ossicles erosion	Cholesteatoma confined to middle ear or mastoid cavity
2	Tegmen tympani erosion and/or Lateral semicircular canal erosion and/or facial nerve canal erosion and/or sigmoid sinus bone plate erosion	Cholesteatoma exceeds middle ear/mastoid cavity, or invades facial nerve

*CT, computed tomography.*

### Microbial Culture

Mycological analysis was carried out in the Microbiology Department of our hospital. Microscopic examination was performed by Zeiss Axiovert 200 under 40×, to initially detect any direct signs of bacterial or fungal infection in the debris. The remaining tissue samples were cultured on Sabouraud’s Dextrose Agar for assessment of fungal growth and on blood Agar and Mac Conkey’s Agar for bacterial growth. Identification of isolated colonies was based on colony morphology, pigmentation, microscopy, and other standard physiological and biochemical assays (Procop et al., 2017).

### DNA Extraction, Metagenomic Sequencing, and Data Analysis

All samples were subjected to DNA extraction (each patient and “no-template” as negative control), library preparation, and next-generation sequencing (NGS). DNA extraction and library preparation were conducted using an NGS Automatic Library Preparation System (Cat. MAR002, MatriDx Biotech Corp., Hangzhou). The reagents included a Nucleic Acid Extraction Kit (Cat. MD013, MatriDx Biotech Corp., Hangzhou) and a Total DNA Library Preparation Kit (other samples) (Cat. MD001T, MatriDx Biotech Corp., Hangzhou). To control the contamination of each sequencing run, each sequencing run on Illumina Next Seq instrument included “no template” as negative control. Quality control was carried out using a bioanalyzer (Agilent 2100, Agilent Technologies, Santa Clara, CA, United States) combined with quantitative PCR to measure the adapters before sequencing. Libraries were pooled and then sequenced on an Illumina NextSeq500 system using a 75-cycle sequencing kit. A total of 10–20 million reads were obtained for each sample.

### Metagenomic Next-Generation Sequencing Data Analysis

High quality sequencing data was generated after removal of short (<35 bp) reads, low quality and low complexity reads. The sequencing data was firstly demultiplexed to get the sequence reads of each sample in fastq format. Then, sequence reads of each sample were aligned to human-specific database constructed from Homo sapiens sequences in NCBI nucleotide (nt) database using bowtie2 (Langmead and Salzberg, 2012; Wood et al., 2019) (version 2.3.5.1) for eliminating the human sequences. The remaining reads were aligned to a manual-curated microbial database using kraken2 (confidence = 0.5) for quickly classification and the classified reads of interested microorganisms were aligned again using bowtie2 for validation. Additionally, the result of microorganism classification was used to identify the potential pathogens.

### Statistical Analysis

Significant differences in the relative abundance of microbial taxa (i.e., the proportions of each microbe) between patient groups (categorized by CT score) were determined using *t*-tests. Fisher’s exact test were used to evaluate independent binomial variables (SPSS version 20). The *p*-value of ≤0.05 was considered statistically significant.

## Results

### Clinical Findings

**FIGURE 1:**
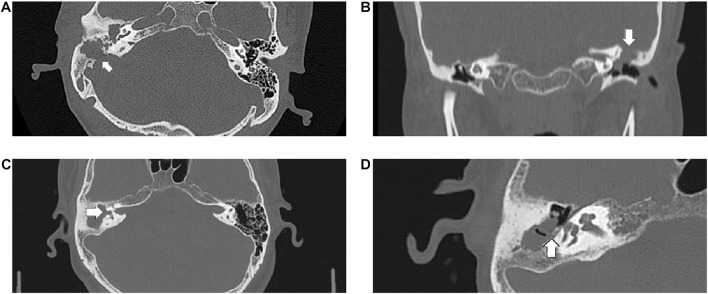
The following CT scan findings were recorded as score 2: **(A)** the sigmoid sinus bone plate eroded (arrowed), **(B)** coronal section of the left epitympanum demonstrating a tegmen tympani defect (arrowed), **(C)** a large defect of the lateral semicircular canal (arrowed), **(D)** the pyramid segment of facial canal eroded (arrowed).

Sixteen patients with cholesteatoma were enrolled in this study. Ten were male and six were female, with an age range of 17 to 75 years. Clinical data are shown in Table 2. All patients showed purulent discharge and hearing loss. Two of them presented with otalgia, and two other patients displayed facial paralysis. Most frequently, pre-operation otoscopic findings were pars flaccida retraction (12/16) and three of them had granulation in the retraction pocket. Pars tensa perforation was found in two patients. The two patients who complained of otalgia had external canal collapse, which precluded observation of the tympanic membrane. Seven patients with severe temporal bone erosion were recorded as CT score 2 (Table 2 and Figure 1).

**TABLE 2:** The demographic data and clinical features of patients with cholesteatoma.

Patients No.	Age (years)	Gender	Affected ear	Clinical presentation	Otoscopic findings	CT scores
1	44	M	R	Pulurent discharge + otalgia + HL	Postero-superior CW collapse	2
2	58	F	L	Pulurent discharge + HL	Pars flaccida retraction	2
3	17	F	R	Pulurent discharge + HL	Pars flaccida retraction	1
4	20	M	L	Pulurent discharge + HL	Pars flaccida retraction + granulation	1
5	21	M	R	Pulurent discharge + HL + FP	Pars flaccida retraction	1
6	75	F	R	Pulurent discharge + HL + FP	Pars tensa perforation	2
7	49	M	R	Pulurent discharge + HL	Pars flaccida retraction	1
8	55	M	L	Pulurent discharge + HL	Pars flaccida retraction + granulation	1
9	64	M	R	Pulurent discharge + HL	Pars flaccida retraction	2
10	62	F	R	Pulurent discharge + HL	Pars tensa perforation + granulation	1
11	74	F	L	Pulurent discharge + HL	Pars flaccida retraction	1
12	45	M	L	Pulurent discharge + otalgia + HL	Postero-superior CW collapse	2
13	43	M	R	Pulurent discharge + HL	Pars flaccida retraction	2
14	23	M	L	Pulurent discharge + HL	Pars flaccida retraction	1
15	57	F	R	Pulurent discharge + HL	Pars flaccida retraction	1
16	72	M	R	Pulurent discharge + HL	Pars flaccida retraction + granulation	2

*No, number; M, male; F, female; R, right; L, left; HL, hearing loss; FP, facial paralysis; CW, canal wall.*

### Microbial Culture Results

Eleven (68.75%) of the 16 patients, were negative for culturable bacteria or fungi (Table 3). In the remaining five patients, bacterial species were successfully cultured and identified in four cholesteatoma specimens, and fungal species were detected in only one specimen. *Staphylococcus* was the most common bacterial species (75%).

**TABLE 3:** Microbiological culture results and mNGS results of 16 cholesteatoma samples.

Patients No.	Microbiological culture	mNGS Organism	Reads	Relative abundance
1	Negative	*Bacteroides fragilis*	551	53.08%

2	Aspergillus flavus	*Aspergillus flavus*	2882	90.34%

3	Negative	*Enterobacter cloacae complex*	6	2.39%

4	Negative	*Candida parapsilosis*	164312	16.52%
		*Mobiluncus curtisii*	45456	4.61%
		*Porphyromonas asaccharolytica*	27033	2.74%
		*Campylobacter ureolyticus*	21601	2.19%
		*Anaerococcus prevotii*	10743	1.09%
		*Staphylococcus*	10485	1.06%
		*Streptococcus pseudoporcinus*	8225	0.83%
		*Finegoldia magna*	6445	0.65%

5	Negative	*Campylobacter ureolyticus*	42466	33.79%
		*Finegoldia magna*	11000	26.49%
		*Enterococcus faecalis*	2873	2.29%

6	Negative	*Aspergillus terreus*	73	15.87%
		*Lactobacillus iners*	86	8.74%

7	Negative	*Staphylococcus aureus*	989	75.44%
		*Aspergillus*	58	4.42%
		*Nocardiopsis dassonvillei*	13	0.99%

8	Proteus mirabilis	*Proteus mirabilis*	48358	39.87%
		*Prevotella oris*	19821	16.34%
		*Parvimonas micra*	8289	6.83%
		*Campylobacter showae*	7451	6.14%
		*Aspergillus*	2123	1.75%

9	Negative	*Aspergillus*	452	20.28%
		*Streptomyces*	277	12.43%
		*Pseudomonas stutzeri*	259	11.62%
		*Nocardiopsis dassonvillei*	87	3.9%

10	Corynebacterium segmentosum	*Corynebacterium segmentosum*	4541295	87.24%
		*Staphylococcus*	241076	4.63%
		*Aspergillus*	8847	0.17%

11	Staphylococcus epidermidis	*Staphylococcus epidermidis*	1435881	90.15%
		*Aspergillus*	792	0.05%
		*Pseudomonas stutzeri*	504	0.03%

12	Negative	*Anaerococcus*	288	24.26%
		*Staphylococcus caprae*	113	9.52%
		*Peptoniphilus harei*	102	8.51%
		*Aspergillus*	6	0.51%

13	Staphylococcus aureus	*Staphylococcus aureus*	37824	90.99%
14	Negative	*Prevotella buccalis*	25447	31.99%
		*Anaerococcus hydrogenalis*	15306	19.24%
		*Pseudomonas aeruginosa*	6468	8.13%
		*Finegoldia magna*	4354	5.47%
		*Trichophyton rubrum*	285	0.36%
		*Aspergillus*	1	0.01%

15	Negative	*Aspergillus*	1	2.08%

16	Negative	*Staphylococcus caprae*	110286	64.73%
		*Corynebacterium jeikeium*	34988	20.54%
		*Aspergillus*	14	0.01%

### Metagenomic Next-Generation Sequencing Results

**FIGURE 2:**
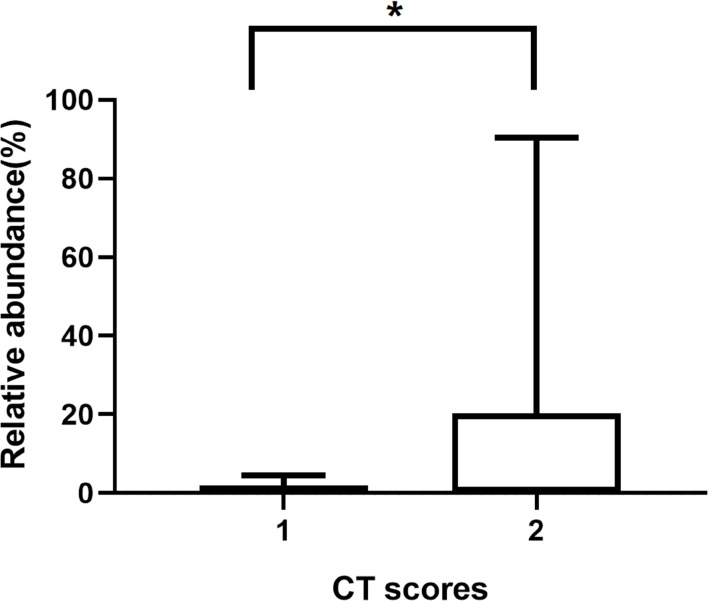
The relative abundances of *Aspergillus* in patients with different CT scores. *p ≤ 0.05.

Our mNGS reads showed a 100% detection rate of potential pathogens in all cholesteatoma samples (Table 3). After excluding the microbial taxa that obviously originated from the environment or a background source, a total of 31 microbial species, including fungi and bacteria were recognized. Eleven samples (68.75%) had more than two different species, and cholesteatoma-specific microbiota were comprised of 2 to 8 taxa. The most commonly detected microbial genus was *Aspergillus* (11/16, 68.75%), with a huge variation in read counts (1 to 2882) and relative abundances (0.01% to 90.34%) (Table 3). The second most common genus was *Staphylococcus* (7/16, 43.75%), with the reads counts ranging from 113 to 1435881 and relative abundances between 1.06% and 90.99% across samples in which was detected (Table 3). The relative abundances of *Aspergillus* significantly varied between patient groups. Patients with the high CT scores have significantly higher relative abundances of *Aspergillus* than those with low CT scores (*p* = 0.023) (Table 4 and Figure 2).

**TABLE 4:** Relative abundances of *Aspergillus* in patients with different CT scores.

CT scores	Patients No.	Relative abundances (%)	Reads
2	1	0	0
	2	90.34	2882
	6	15.87	73
	9	20.28	452
	12	0.51	6
	13	0	0
	16	0.01	14

1	3	0	0
	4	0	0
	5	0	0
	7	4.42	58
	8	1.75	2123
	10	0.17	8847
	11	0.05	792
	14	0.01	1
	15	2.08	1

*CT, computed tomography.*

## Discussion

Several studies have used mNGS as a tool for universal pathogen detection in infectious disease, thus indicating its potential in clinical diagnoses, particularly in microbiota-associated disorders (Hu et al., 2018; Zhu et al., 2018). The advantages of mNGS sequence-based detection of microbes associated with a disease state include high throughput evaluation that can accommodate many samples at once compared to traditional PCR-based detection, the quantity of data provided by sequence is potentially much higher because bacterial, fungal, and viral community members can be detected simultaneously, and the reliability of sequence data surpasses that of traditional morphological and physiological assays (i.e., Biolog plates) for microbial identification (Jacob, 2013).

It is noteworthy that mNGS successfully identified pathogenic fungi or bacteria in all cases, whereas traditional culture-based identification found pathogenic microbes in only 31.25% of our cholesteatoma cohort. The presence and high relative abundance of microbes found by culture-based methods was confirmed by mNGS data, thereby illustrating the suitability of mNGS for detection of pathogens in cholesteatoma debris. However, the two techniques differed in the most commonly detected microbes, since culture methods showed *Staphylococcus* as a predominant taxon, while mNGS showed *Aspergillus* as the most common pathogen associated with cholesteatoma. Besides, *Staphylococcus* was still the second most common pathogens by mNGS. These mNGS results thus offer a new perspective on the effects incurred by microbiota on the development of cholesteatoma.

Standard textbook descriptions of cholesteatoma have historically included bacteria associated with this disorder, but have almost completely neglected the fungal constituents of the debris. Previous studies of chronic rhinosinusitis biofilms have revealed the presence of fungal constituents (Healy et al., 2008), alternatively, fungal colonization of cholesteatoma had been sparsely studied. Especially two studies cultured the fungi from cholesteatoma tissue (Effat and Madany, 2014; Singh et al., 2018). Singh et al. (2018) cultured 40 cholesteatoma samples and successfully isolated fungi from 17 of the specimens (42.5%). Among these 17 cases, 6 cases had detectable grown of both fungi and bacteria. Effat and Madany (2014) obtained keratinous debris from 18 patients (19 ears) with cholesteatoma and found culturable fungi in 17 of the samples (89%), but reported no data on culturable bacterial. More recently, Weiss et al. (2019) evaluated the microbiota associated with cholesteatoma by 16S rDNA gene sequencing in 19 patients. A total of 96 bacterial species and 34 fungal species were identified in association with the cholesteatoma matrix, but no difference was found between the relative abundance of fungal and bacterial taxa prevalent in the diseased tissue compared with that of the adjacent, uninvolved middle ear.

In our study, we used mNGS to analyze the cholesteatoma microbiome for the presence of pathogens. In contrast with conventional culture methods and 16S rDNA gene sequencing, mNGS offers higher sensitivity and throughput microbial pathogen detection and microbiome analyses due to its capacity for simultaneous detection of bacteria and fungi. In our case series, a total of 12 cases (12/16, 75%) exhibited detectable fungal infection, among which 10 cases (10/16, 62.5%) had both bacterial and fungal microbes, a substantially higher proportion than that reported through culture-based investigation (6/17, 35.3%) (Singh et al., 2018). The common fungal genus specifically associated with cholesteatoma in our cholesteatoma cohort was *Aspergillus*, differing from the consistent prevalence of *Alternaria* reported by Weiss et al. (2019). Moreover, only two cases in this study had *Aspergillus* in the absence of any significant bacterial taxa.

As discussed above, fungi have been found to colonize bacterial biofilms in chronic rhinosinusitis. In cholesteatomas, bacterial biofilms were also detected by electron microscopy (Saunders et al., 2011). In the current study, both bacterial and fungal growth was detected in more than half of the samples, consistent with that fungi also likely reside in bacterial biofilms related to cholesteatomas. Some studies have investigated the interactions between bacteria and fungi and their impact on the efficacy of antimicrobial agents. In chronic rhinosinusitis, for example, fungi and bacteria were found to act synergistically against host defenses and antibiotics (Healy et al., 2008). Studies exploring the mechanism underpinning this phenomenon proposed that fungi could interact with bacteria to become coated in the biofilm matrix which subsequently served as a barrier preventing diffusion of antimicrobial agents and facilitating the secretion and diffusion of polysaccharides by fungal cells (Harriott and Noverr, 2010; Kong et al., 2016). The interaction between fungi and bacteria may be also associated with poor response to antibiotic therapy in cholesteatoma. In future studies, we will explore the mechanism behind this phenomenon of the relationship between *Aspergillus* and cholesteatoma.

A study by Kauffman et al. (2000) using airway-derived epithelial cell line A549 and primary epithelial nasal cells found that exposure to *Aspergillus* extracts resulted in cell shrinking, cell desquamation, and proinflammatory cytokine production. More recently, Patel et al. (2019) demonstrated that *Aspergillus* could stimulate IL-25 secretion and elicit type 2 inflammation in non-invasive fungal rhinosinusitis. It is therefore plausible that *Aspergillus* could play a role in cholesteatoma progression. To explore this possibility in our study, we used CT scans to evaluate bone erosion related to cholesteatoma and found that *Aspergillus* infection was identified more often in cases with severe bone erosion, compared with its prevalence in cases with minor bone erosion. Similarly, in allergic fungal rhinosinusitis, *Aspergillus* was also reportedly associated with bone destruction (Marfani et al., 2010). Many studies have focused on osteoprotegerin (OPG), a receptor activator of nuclear factor κB (RANK) and RANK ligand (RANKL), in cholesteatoma-related bone erosion (Jeong et al., 2006; Kuczkowski et al., 2010; Likus et al., 2016). However, some inconsistencies remain in the relationship between these proteins and cholesteatoma. Specifically, although inflammatory cytokines and bacteria were thought to be correlated with OPG and RANKL expression and bone destruction in cholesteatoma (Haruyama et al., 2010; Kalcioglu et al., 2018), no significant differences were observed in the expression of OPG and RANKL between the cholesteatoma cases with and without bacterial infections (Kalcioglu et al., 2018). Thus, further study the role of fungi and bacteria in bone erosion in cholesteatoma is warranted.

In conclusion, this study represents the first report to our knowledge using mNGS to detect microbes in cholesteatoma samples. It is noteworthy that the most commonly detected microbe specifically associated with cholesteatoma is *Aspergillus* and its presence is apparently related to increase the degree of bone destruction. This study had some limitations, such as relatively small sample size and use of CT scoring to differentiate disease states instead of healthy control subjects. In addition, the read counts varied greatly between samples. It is possible that water lavage during oto-surgery may have resulted in the loss or reduction in microbial load during surgery. To accommodate this issue in the present study, the cut-off value for potential pathogen detection was set low and at least one unique read was used as a threshold. However, similar methods have been used by other mNGS studies (Miao et al., 2018; Wang et al., 2019). Regardless of its limitations, the results provided by this mNGS analysis likely represent the most accurate characterization of the cholesteatoma microbiota to date and provide insight into how microbial taxa may contribute to the severity of this disease.

## Data Availability Statement

The datasets presented in this study can be found in online repositories. The names of the repository/repositories and accession number(s) can be found below: NCBI (accession: PRJNA768391).

## Ethics Statement

The studies involving human participants were reviewed and approved by The Second Affiliated Hospital, Zhejiang University School of Medicine. The patients/participants provided their written informed consent to participate in this study. Written informed consent was obtained from the individual(s) for the publication of any potentially identifiable images or data included in this article.

## Author Contributions

XC and HJ conceived and designed the study. XC, HJ, and LH analyzed the data. HJ, QW, LS, XO, and HL collected the related clinical information. HJ, CW, JX, XH, JW, and WD conducted the infection samples associated with the study. XH, JW, and WD provided the technical support. XC and HJ wrote the draft. LH, JX, and CW revised the draft. All authors approved the final version. All authors contributed to the article and approved the submitted version.

## Conflict of Interest

XH, JW, and WD are employed by “Matridx Biotechnology Co., Ltd.”

The remaining authors declare that the research was conducted in the absence of any commercial or financial relationships that could be construed as a potential conflict of interest.

## Publisher’s Note

All claims expressed in this article are solely those of the authors and do not necessarily represent those of their affiliated organizations, or those of the publisher, the editors and the reviewers. Any product that may be evaluated in this article, or claim that may be made by its manufacturer, is not guaranteed or endorsed by the publisher.

## References

[B1] Effat K. Madany N. (2014). Mycological study on cholesteatoma keratin obtained during primary mastoid surgery. *J. Laryngol. Otol.* 128:881. 10.1017/S0022215114002059 25236688

[B2] Fan S. Ren H. Wei Y. Mao C. Ma Z. Zhang L. (2018). Next-generation sequencing of the cerebrospinal fluid in the diagnosis of neurobrucellosis. *Int. J. Infect. Dis.* 67 20–24. 10.1016/j.ijid.2017.11.028 29196276

[B3] Harriott M. M. Noverr M. C. (2010). Ability of Candida albicans mutants to induce Staphylococcus aureus vancomycin resistance during polymicrobial biofilm formation. *Antimicrob. Agents Chemother.* 54 3746–3755. 10.1128/AAC.00573-10 20566760 PMC2934986

[B4] Haruyama T. Furukawa M. Kusunoki T. Onoda J. Ikeda K. (2010). Expression of IL-17 and its role in bone destruction in human middle ear cholesteatoma. *ORL J. Otorhinolaryngol. Relat. Spec.* 72 325–331. 10.1159/000319897 20847582

[B5] Healy D. Y. Leid J. G. Sanderson A. R. Hunsaker D. H. (2008). Biofilms with fungi in chronic rhinosinusitis. *Otolaryngol. Head Neck Surg.* 138 641–647. 10.1016/j.otohns.2008.02.002 18439472

[B6] Hu Z. Weng X. Xu C. Lin Y. Cheng C. Wei H. (2018). Metagenomic next-generation sequencing as a diagnostic tool for toxoplasmic encephalitis. *Ann. Clin. Microbiol. Antimicrob.* 17:45. 10.1186/s12941-018-0298-1 30587202 PMC6305995

[B7] Inagaki T. Paparella M. M. (2009). Chronic otitis media with cholesteatoma: middle ear/inner ear interaction. *Otol. Neurotol.* 30 430–431. 10.1097/MAO.0b013e31818600db 18800021

[B8] Jacob H. J. (2013). Next-generation sequencing for clinical diagnostics. *N. Engl. J. Med.* 369 1557–1558. 10.1056/NEJMe1310846 24088040

[B9] Jeong J. H. Park C. W. Tae K. Lee S. H. Shin D. H. Kim K. R. (2006). Expression of RANKL and OPG in middle ear cholesteatoma tissue. *Laryngoscope* 116 1180–1184. 10.1097/01.mlg.0000224345.59291.da 16826057

[B10] Kalcioglu M. T. Guldemir D. Unaldi O. Egilmez O. K. Celebi B. Durmaz R. (2018). Metagenomics analysis of bacterial population of tympanosclerotic plaques and cholesteatomas. *Otolaryngol. Head Neck Surg.* 159 724–732. 10.1177/0194599818772039 29688828

[B11] Kauffman H. F. Tomee J. C. Van De Riet M. A. Timmerman A. J. Borger P. (2000). Protease-dependent activation of epithelial cells by fungal allergens leads to morphologic changes and cytokine production. *J. Allergy Clin. Immunol.* 105 1185–1193. 10.1067/mai.2000.106210 10856154

[B12] Kong E. F. Tsui C. Kucharíková S. Andes D. Van Dijck P. Jabra-Rizk M. A. (2016). Commensal protection of Staphylococcus aureus against antimicrobials by Candida albicans biofilm matrix. *MBio* 7 e1365–16. 10.1128/mBio.01365-16 27729510 PMC5061872

[B13] Kuczkowski J. Sakowicz-Burkiewicz M. Iżycka-Świeszewska E. (2010). Expression of the receptor activator for nuclear factor-κB ligand and osteoprotegerin in chronic otitis media. *Am. J. Otolaryngol.* 31 404–409. 10.1016/j.amjoto.2009.06.004 20015790

[B14] Langmead B. Salzberg S. L. (2012). Fast gapped-read alignment with Bowtie 2. *Nat. Methods* 9:357. 10.1038/nmeth.1923 22388286 PMC3322381

[B15] Likus W. Siemianowicz K. Markowski J. Wiaderkiewicz J. Kostrza̧b-Zdebel A. Jura-Szołtys E. (2016). Bacterial infections and osteoclastogenesis regulators in men and women with cholesteatoma. *Arch. Immunol. Ther. Exp.* 64 241–247. 10.1007/s00005-015-0373-7 26584851

[B16] Liu C. M. Cosetti M. K. Aziz M. Buchhagen J. L. Contente-Cuomo T. L. Price L. B. (2011). The otologic microbiome: a study of the bacterial microbiota in a pediatric patient with chronic serous otitis media using 16SrRNA gene-based pyrosequencing. *Arch. Otolaryngol. Head Neck Surg.* 137 664–668. 10.1001/archoto.2011.116 21768410

[B17] Mai N. T. H. Phu N. H. Nhu L. N. T. Hong N. T. T. Hanh N. H. H. Nguyet L. A. (2017). Central nervous system infection diagnosis by next-generation sequencing: a glimpse into the future?. *Open Forum Infect. Dis.* 4:ofx046. 10.1093/ofid/ofx046 28480297 PMC5411956

[B18] Marfani M. Jawaid M. Shaikh S. Thaheem K. (2010). Allergic fungal rhinosinusitis with skull base and orbital erosion. *J. Laryngol. Otol.* 124:161. 10.1017/S0022215109991253 19954557

[B19] Miao Q. Ma Y. Wang Q. Pan J. Zhang Y. Jin W. (2018). Microbiological diagnostic performance of metagenomic next-generation sequencing when applied to clinical practice. *Clin. Infect. Dis.* 67 S231–S240. 10.1093/cid/ciy693 30423048

[B20] Parisier S. C. (1989). Management of cholesteatoma. *Otolaryngol. Clin. North Am.* 22 927–940. 10.1016/S0030-6665(20)31368-2 2616169

[B21] Patel N. N. Triantafillou V. Maina I. W. Workman A. D. Tong C. C. Kuan E. C. (2019). Fungal extracts stimulate solitary chemosensory cell expansion in noninvasive fungal rhinosinusitis. *Int. Forum Allergy Rhinol.* 9 730–737. 10.1002/alr.22334 30892837 PMC6640101

[B22] Procop G. W. Church D. Hall G. Janda W. (2017). *Koneman’s Color Atlas and Textbook of Diagnostic.* Philadelphia: Wolters Kluwer.

[B23] Ricciardiello F. Cavaliere M. Mesolella M. Iengo M. (2009). Notes on the microbiology of cholesteatoma: clinical findings and treatment. *Acta Otorhinolaryngol. Ital.* 29:197. PMC2816367 20161877

[B24] Saunders J. Murray M. Alleman A. (2011). Biofilms in chronic suppurative otitis media and cholesteatoma: scanning electron microscopy findings. *Am. J. Otolaryngol.* 32 32–37. 10.1016/j.amjoto.2009.09.010 20036033

[B25] Singh G. B. Solo M. Kaur R. Arora R. Kumar S. (2018). Mycology of chronic suppurative otitis media-cholesteatoma disease: an evaluative study. *Am. J. Otolaryngol.* 39 157–161. 10.1016/j.amjoto.2017.12.001 29306568

[B26] Wang S. Chen Y. Wang D. Wu Y. Zhao D. Zhang J. (2019). The feasibility of metagenomic next-generation sequencing to identify pathogens causing tuberculous meningitis in cerebrospinal fluid. *Front. Microbiol.* 10:1993. 10.3389/fmicb.2019.01993 31551954 PMC6733977

[B27] Weiss J. P. Antonelli P. J. Dirain C. O. (2019). Microbiome Analysis of Cholesteatoma by Gene Sequencing. *Otol. Neurotol.* 40 1186–1193. 10.1097/MAO.0000000000002355 31469791

[B28] Welkoborsky H. (2011). Current concepts of the pathogenesis of acquired middle ear cholesteatoma. *Laryngorhinootologie* 90 38–48. 10.1055/s-0030-1268464 21225533

[B29] Wilson M. R. Suan D. Duggins A. Schubert R. D. Khan L. M. Sample H. A. (2017). A novel cause of chronic viral meningoencephalitis: cache Valley virus. *Ann. Neurol.* 82 105–114. 10.1002/ana.24982 28628941 PMC5546801

[B30] Wood D. E. Lu J. Langmead B. (2019). Improved metagenomic analysis with Kraken 2. *Genome Biol.* 20:257. 10.1186/s13059-019-1891-0 31779668 PMC6883579

[B31] Zhu Y.-M. Ai J.-W. Xu B. Cui P. Cheng Q. Wu H. (2018). Rapid and precise diagnosis of disseminated T. marneffei infection assisted by high-throughput sequencing of multifarious specimens in a HIV-negative patient: a case report. *BMC Infect. Dis.* 18:379. 10.1186/s12879-018-3276-5 30086724 PMC6081951

