# Supplementary material for: Bacterial and Fungal Infections Promote the Bone Erosion Progression in Acquired Cholesteatoma Revealed by Metagenomic Next-Generation Sequencing
Source: Front Microbiol. 2021 Nov 5;12:761111. doi: 10.3389/fmicb.2021.761111 (PMC8604023; doi:10.3389/fmicb.2021.761111)
Supplement: Supplementary file 1 [file Data_Sheet_1.PDF]

Patient No.	Organism	Reads	RPM
1	Bacteroides	551	63.24
	Bacteroides fragilis	407	46.71
	Bacteroides thetaiotaomicron	107	12.28
	Bacteroides ovatus	2	0.23
	Bacteroides uniformis	1	0.11
	Porphyromonas	87	9.98
	Porphyromonas gingivalis	79	9.07
	Porphyromonas asaccharolytica	8	0.92
	Citrobacter	77	8.84
	Citrobacter farmeri	55	6.31
	Citrobacter amalonaticus	7	0.8
	Arcobacter	42	4.82
	Arcobacter butzleri	41	4.71
	Tannerella	40	4.59
	Tannerella forsythia	40	4.59
	Fusobacterium	30	3.44
	Fusobacterium nucleatum	20	2.3
	Burkholderia	19	2.18
	Burkholderia cepacia complex	18	2.07
	Burkholderia contaminans	13	1.49
	Mobiluncus	6	0.69
	Mobiluncus curtisii	6	0.69
	Ralstonia	5	0.57
	Ralstonia pickettii	5	0.57
	Staphylococcus	3	0.34
	Staphylococcus aureus	1	0.11
	Staphylococcus warneri	1	0.11
	Acinetobacter	2	0.23
	Acinetobacter johnsonii	1	0.11
	Pseudomonas	2	0.23
	Pseudomonas stutzeri	2	0.23
	Kocuria	2	0.23
	Kocuria palustris	1	0.11
	Kocuria rosea	1	0.11
	Corynebacterium	2	0.23
	Corynebacterium jeikeium	2	0.23
	Cutibacterium	2	0.23
	Cutibacterium acnes	2	0.23
	Prevotella	1	0.11
	Odoribacter	1	0.11
	Odoribacter splanchnicus	1	0.11
	Streptococcus	1	0.11
	Streptococcus pneumoniae	1	0.11
	Lactococcus	1	0.11
	Lactococcus lactis	1	0.11

Patient No.	Organism	Reads	RPM
	Micrococcus	1	0.11
	Micrococcus luteus	1	0.11
	Rothia	1	0.11
	Yarrowia	1	0.11
	Yarrowia lipolytica	1	0.11
2	Cutibacterium	55	7.84
	Cutibacterium acnes	55	7.84
	Burkholderia	37	5.28
	Burkholderia cepacia complex	20	2.85
	Burkholderia vietnamiensis	10	1.43
	Burkholderia contaminans	4	0.57
	Corynebacterium	29	4.14
	Corynebacterium striatum	25	3.57
	Corynebacterium matruchotii	2	0.29
	Acinetobacter	17	2.42
	Acinetobacter johnsonii	7	1
	Acinetobacter schindleri	1	0.14
	Acinetobacter haemolyticus	1	0.14
	Prevotella	14	2
	Prevotella intermedia	8	1.14
	Prevotella melaninogenica	3	0.43
	Prevotella scopos	2	0.29
	Prevotella denticola	1	0.14
	Moraxella	9	1.28
	Moraxella osloensis	9	1.28
	Staphylococcus	9	1.28
	Staphylococcus epidermidis	2	0.29
	Staphylococcus haemolyticus	1	0.14
	Staphylococcus hominis	1	0.14
	Staphylococcus capitis	1	0.14
	Micrococcus	8	1.14
	Micrococcus luteus	8	1.14
	Ralstonia	5	0.71
	Ralstonia pickettii	5	0.71
	Porphyromonas	4	0.57
	Porphyromonas gingivalis	4	0.57
	Chryseobacterium	3	0.43
	Pseudomonas	2	0.29
	Veillonella	2	0.29
	Veillonella parvula	2	0.29
	Kocuria	2	0.29
	Kocuria rosea	2	0.29
	Microbacterium	2	0.29
	Microbacterium oxydans	1	0.14
	Nocardiopsis	2	0.29

Patient No.	Organism	Reads	RPM
	Nocardiopsis dassonvillei	2	0.29
	Klebsiella	1	0.14
	Klebsiella aerogenes	1	0.14
	Streptococcus	1	0.14
	Streptococcus anginosus	1	0.14
	Enterococcus	1	0.14
	Enterococcus faecalis	1	0.14
	Aspergillus	2882	411.01
	Aspergillus flavus	2259	322.16
	Aspergillus oryzae	159	22.68
	Aspergillus nomius	12	1.71
	Aspergillus bombycis	11	1.57
	Aspergillus niger	1	0.14
	Aspergillus fischeri	1	0.14
	Aspergillus terreus	1	0.14
	Yarrowia	1	0.14
	Yarrowia lipolytica	1	0.14
	Alternaria	1	0.14
	Alternaria alternata	1	0.14
3	Burkholderia	66	8.56
	Burkholderia cepacia complex	63	8.17
	Burkholderia contaminans	55	7.14
	Ralstonia	8	1.04
	Ralstonia pickettii	8	1.04
	Enterobacter	6	0.78
	Enterobacter cloacae complex	6	0.78
	Enterobacter ludwigii	1	0.13
	Cutibacterium	4	0.52
	Cutibacterium acnes	4	0.52
	Stenotrophomonas	2	0.26
	Stenotrophomonas maltophilia	2	0.26
	Acinetobacter	1	0.13
	Acinetobacter johnsonii	1	0.13
	Moraxella	1	0.13
	Moraxella osloensis	1	0.13
	Pseudomonas	1	0.13
	Methylobacterium	1	0.13
	Sphingomonas	1	0.13
	Sphingomonas paucimobilis	1	0.13
	Kocuria	1	0.13
	Kocuria rosea	1	0.13
4	Mobiluncus	45456	4649.95
	Mobiluncus curtisii	45426	4646.88

Patient No.	Organism	Reads	RPM
	Porphyromonas	27033	2765.36
	Porphyromonas asaccharolytica	26486	2709.4
	Porphyromonas gingivalis	209	21.38
	Porphyromonas uenonis	19	1.94
	Campylobacter	21601	2209.69
	Campylobacter ureolyticus	13651	1396.44
	Campylobacter showae	3209	328.27
	Campylobacter hominis	829	84.8
	Campylobacter fetus	4	0.41
	Campylobacter concisus	2	0.2
	Campylobacter jejuni	1	0.1
	Campylobacter curvus	1	0.1
	Campylobacter iguaniorum	1	0.1
	Anaerococcus	10743	1098.96
	Anaerococcus prevotii	1842	188.43
	Staphylococcus	10485	1072.57
	Staphylococcus lugdunensis	5033	514.85
	Staphylococcus aureus	4781	489.08
	Staphylococcus epidermidis	433	44.29
	Staphylococcus saprophyticus	3	0.31
	Staphylococcus cohnii	2	0.2
	Staphylococcus hominis	2	0.2
	Staphylococcus warneri	2	0.2
	Staphylococcus capitis	1	0.1
	Staphylococcus haemolyticus	1	0.1
	Staphylococcus hyicus	1	0.1
	Streptococcus	8225	841.38
	Streptococcus pseudoporcinus	2676	273.74
	Streptococcus pyogenes	1404	143.62
	Streptococcus agalactiae	919	94.01
	Streptococcus dysgalactiae	401	41.02
	Streptococcus anginosus	269	27.52
	Streptococcus equi	193	19.74
	Streptococcus pneumoniae	139	14.22
	Streptococcus constellatus	26	2.66
	Streptococcus intermedius	7	0.72
	Streptococcus mitis	7	0.72
	Streptococcus suis	4	0.41
	Streptococcus oralis	1	0.1
	Finegoldia	6445	659.3
	Finegoldia magna	6445	659.3
	Corynebacterium	4825	493.58
	Corynebacterium striatum	105	10.74
	Corynebacterium diphtheriae	74	7.57

Patient No.	Organism	Reads	RPM
	Corynebacterium simulans	14	1.43
	Corynebacterium resistens	7	0.72
	Corynebacterium renale	7	0.72
	Corynebacterium accolens	6	0.61
	Corynebacterium jeikeium	4	0.41
	Corynebacterium ureicelerivorans	3	0.31
	Corynebacterium camporealensis	2	0.2
	Corynebacterium riegelii	1	0.1
	Corynebacterium aurimucosum	1	0.1
	Prevotella	4775	488.46
	Prevotella intermedia	1141	116.72
	Prevotella scopos	398	40.71
	Prevotella enoeca	251	25.68
	Prevotella fusca	234	23.94
	Prevotella melaninogenica	157	16.06
	Prevotella dentalis	132	13.5
	Prevotella denticola	123	12.58
	Prevotella bivia	84	8.59
	Prevotella oris	57	5.83
	Prevotella timonensis	9	0.92
	Prevotella buccalis	6	0.61
	Prevotella loescheii	2	0.2
	Prevotella ruminicola	1	0.1
	Prevotella buccae	1	0.1
	Peptoniphilus	4504	460.74
	Peptoniphilus harei	4018	411.02
	Peptoniphilus lacrimalis	37	3.78
	Peptoniphilus asaccharolyticus	4	0.41
	Aerococcus	1107	113.24
	Aerococcus urinae	1054	107.82
	Aerococcus christensenii	53	5.42
	Bifidobacterium	1104	112.93
	Bifidobacterium longum	1096	112.12
	Bifidobacterium breve	8	0.82
	Bacteroides	1094	111.91
	Bacteroides fragilis	684	69.97
	Bacteroides heparinolyticus	119	12.17
	Bacteroides zoogleoformans	28	2.86
	Bacteroides thetaiotaomicron	20	2.05
	Phocaeicola vulgatus	5	0.51
	Fastidiosipila	493	50.43
	Fastidiosipila sanguinis	493	50.43
	Fusobacterium	344	35.19
	Fusobacterium periodonticum	37	3.78

Patient No.	Organism	Reads	RPM
	Fusobacterium necrophorum	30	3.07
	Fusobacterium nucleatum	9	0.92
	Mogibacterium	236	24.14
	Mogibacterium pumilum	234	23.94
	Mogibacterium diversum	2	0.2
	Neisseria	118	12.07
	Neisseria polysaccharea	117	11.97
	Neisseria gonorrhoeae	1	0.1
	Gemella	85	8.7
	Gemella haemolysans	85	8.7
	Burkholderia	70	7.16
	Burkholderia cepacia complex	68	6.96
	Burkholderia contaminans	54	5.52
	Burkholderia cenocepacia	3	0.31
	Helcococcus	62	6.34
	Helcococcus kunzii	62	6.34
	Capnocytophaga	47	4.81
	Capnocytophaga ochracea	7	0.72
	Filifactor	44	4.5
	Filifactor alocis	44	4.5
	Ornithobacterium	30	3.07
	Ornithobacterium rhinotracheale	30	3.07
	Odoribacter	21	2.15
	Odoribacter splanchnicus	21	2.15
	Haemophilus	19	1.94
	[Haemophilus] ducreyi	16	1.64
	Haemophilus influenzae	3	0.31
	Erysipelothrix	14	1.43
	Erysipelothrix rhusiopathiae	14	1.43
	Parvimonas	14	1.43
	Parvimonas micra	14	1.43
	Ralstonia	13	1.33
	Ralstonia pickettii	12	1.23
	Streptobacillus	11	1.13
	Streptobacillus moniliformis	11	1.13
	Alistipes	11	1.13
	Alistipes shahii	11	1.13
	Riemerella	7	0.72
	Riemerella anatipestifer	7	0.72
	Gardnerella	7	0.72
	Gardnerella vaginalis	7	0.72
	Parabacteroides	6	0.61
	Parabacteroides distasonis	6	0.61
	Arcanobacterium	6	0.61

Patient No.	Organism	Reads	RPM
	Arcanobacterium haemolyticum	6	0.61
	Enterococcus	5	0.51
	Enterococcus faecium	5	0.51
	Treponema	4	0.41
	Treponema pedis	4	0.41
	Tannerella	4	0.41
	Tannerella forsythia	4	0.41
	Clostridioides	4	0.41
	Clostridioides difficile	4	0.41
	Exiguobacterium	3	0.31
	Exiguobacterium sp. AT1b	2	0.2
	Cutibacterium	3	0.31
	Cutibacterium acnes	3	0.31
	Pseudomonas	1	0.1
	Pseudomonas aeruginosa	1	0.1
	Stenotrophomonas	1	0.1
	Dialister	1	0.1
	Dialister pneumosintes	1	0.1
	Kocuria	1	0.1
	Kocuria palustris	1	0.1
	Candida	164312	16808.39
	Candida parapsilosis	162879	16661.8
	Candida orthopsilosis	64	6.55
	Candida albicans	4	0.41
	Candida tropicalis	3	0.31
	Candida dubliniensis	1	0.1
	Lodderomyces	1	0.1
	Lodderomyces elongisporus	1	0.1
	Trichomonas	90	9.21
	Trichomonas vaginalis	90	9.21
5	Campylobacter	42466	4366.8
	Campylobacter ureolyticus	33287	3422.92
	Campylobacter showae	2646	272.09
	Campylobacter fetus	51	5.24
	Campylobacter hominis	34	3.5
	Finegoldia	11000	1131.14
	Finegoldia magna	11000	1131.14
	Anaerococcus	7021	721.97
	Anaerococcus prevotii	945	97.17
	Peptoniphilus	5508	566.39
	Peptoniphilus harei	3044	313.02
	Peptoniphilus rhinitidis	1761	181.08
	Streptococcus	4713	484.64
	Streptococcus agalactiae	1243	127.82

Patient No.	Organism	Reads	RPM
	Streptococcus pyogenes	668	68.69
	Streptococcus dysgalactiae	305	31.36
	Streptococcus pseudoporcinus	246	25.3
	Streptococcus anginosus	132	13.57
	Streptococcus equi	60	6.17
	Streptococcus pneumoniae	58	5.96
	Streptococcus salivarius	40	4.11
	Streptococcus constellatus	24	2.47
	Streptococcus porcinus	9	0.93
	Streptococcus canis	6	0.62
	Streptococcus suis	3	0.31
	Streptococcus intermedius	2	0.21
	Enterococcus	2873	295.43
	Enterococcus faecalis	2837	291.73
	Enterococcus casseliflavus	1	0.1
	Escherichia	2008	206.48
	Escherichia coli	1942	199.7
	Prevotella	1429	146.94
	Prevotella intermedia	555	57.07
	Prevotella bivia	136	13.98
	Prevotella dentalis	55	5.66
	Prevotella scopos	47	4.83
	Prevotella denticola	27	2.78
	Prevotella melaninogenica	23	2.37
	Prevotella fusca	8	0.82
	Prevotella stercorea	8	0.82
	Prevotella enoeca	7	0.72
	Prevotella oris	7	0.72
	Eikenella	742	76.3
	Eikenella corrodens	731	75.17
	Dermabacter	623	64.06
	Dermabacter hominis	148	15.22
	Schaalia	531	54.6
	Schaalia meyeri	124	12.75
	Schaalia cardiffensis	2	0.21
	Klebsiella	244	25.09
	Klebsiella pneumoniae	180	18.51
	Fastidiosipila	121	12.44
	Fastidiosipila sanguinis	121	12.44
	Blautia	116	11.93
	[Ruminococcus] gnavus	97	9.97
	Gemella	100	10.28
	Gemella haemolysans	100	10.28
	Porphyromonas	56	5.76

Patient No.	Organism	Reads	RPM
	Porphyromonas gingivalis	46	4.73
	Porphyromonas asaccharolytica	10	1.03
	Fusobacterium	53	5.45
	Fusobacterium necrophorum	53	5.45
	Burkholderia	45	4.63
	Burkholderia cepacia complex	42	4.32
	Burkholderia contaminans	39	4.01
	Haemophilus	41	4.22
	Haemophilus influenzae	34	3.5
	Treponema	39	4.01
	Treponema phagedenis	31	3.19
	Treponema pedis	8	0.82
	Actinomyces	33	3.39
	Pauljensenia hongkongensis	31	3.19
	Lactobacillus	32	3.29
	Lactobacillus iners	32	3.29
	Filifactor	28	2.88
	Filifactor alocis	28	2.88
	Erysipelothrix	21	2.16
	Erysipelothrix rhusiopathiae	21	2.16
	Clostridioides	18	1.85
	Clostridioides difficile	18	1.85
	Ralstonia	17	1.75
	Ralstonia pickettii	16	1.65
	Helcococcus	15	1.54
	Helcococcus kunzii	15	1.54
	Eubacterium	13	1.34
	Eubacterium limosum	13	1.34
	Neisseria	12	1.23
	Neisseria elongata	6	0.62
	Neisseria gonorrhoeae	1	0.1
	Neisseria mucosa	1	0.1
	Mogibacterium	10	1.03
	Mogibacterium diversum	8	0.82
	Mogibacterium pumilum	2	0.21
	Dolosigranulum	9	0.93
	Dolosigranulum pigrum	9	0.93
	Parvimonas	8	0.82
	Parvimonas micra	8	0.82
	Aerococcus	7	0.72
	Aerococcus christensenii	5	0.51
	Aerococcus urinae	2	0.21
	Corynebacterium	4	0.41
	Corynebacterium jeikeium	1	0.1

Patient No.	Organism	Reads	RPM
	Cutibacterium	4	0.41
	Cutibacterium acnes	4	0.41
	Acinetobacter	3	0.31
	Acinetobacter johnsonii	1	0.1
	Micrococcus	2	0.21
	Micrococcus luteus	1	0.1
	Rhodococcus	2	0.21
	Shigella	1	0.1
	Shigella dysenteriae	1	0.1
	Providencia	1	0.1
	Providencia rettgeri	1	0.1
	Vibrio	1	0.1
	Vibrio parahaemolyticus	1	0.1
	Comamonas	1	0.1
	Comamonas terrigena	1	0.1
	Clostridium	1	0.1
	Clostridium botulinum	1	0.1
	Rothia	1	0.1
	Rothia mucilaginosa	1	0.1
	Kocuria	1	0.1
	Kocuria palustris	1	0.1
	Kytococcus	1	0.1
	Kytococcus sedentarius	1	0.1
	Pseudopropionibacterium	1	0.1
	Pseudopropionibacterium propionicum	1	0.1
	Alphapapillomavirus	1	0.1
	Alphapapillomavirus 8	1	0.1
	Trichomonas	14	1.44
	Trichomonas vaginalis	14	1.44
6	Lactobacillus	86	8.74
	Lactobacillus iners	85	8.64
	Cutibacterium	62	6.3
	Cutibacterium acnes	62	6.3
	Staphylococcus	23	2.34
	Staphylococcus epidermidis	11	1.12
	Staphylococcus saprophyticus	3	0.31
	Staphylococcus capitis	2	0.2
	Staphylococcus aureus	1	0.1
	Staphylococcus haemolyticus	1	0.1
	Staphylococcus hominis	1	0.1
	Staphylococcus lugdunensis	1	0.1
	Pseudomonas	10	1.02
	Pseudomonas oleovorans	1	0.1
	Pseudomonas monteilii	1	0.1

Patient No.	Organism	Reads	RPM
	Burkholderia	9	0.92
	Burkholderia cepacia complex	5	0.51
	Burkholderia vietnamiensis	5	0.51
	Micrococcus	9	0.92
	Micrococcus luteus	6	0.61
	Acinetobacter	8	0.81
	Acinetobacter johnsonii	4	0.41
	Acinetobacter junii	1	0.1
	Acinetobacter schindleri	1	0.1
	Corynebacterium	8	0.81
	Corynebacterium segmentosum	4	0.41
	Corynebacterium matruchotii	2	0.2
	Moraxella	7	0.71
	Moraxella osloensis	7	0.71
	Streptococcus	5	0.51
	Streptococcus oralis	1	0.1
	Streptococcus pneumoniae	1	0.1
	Veillonella	5	0.51
	Veillonella parvula	5	0.51
	Actinomyces	5	0.51
	Actinomyces naeslundii	1	0.1
	Actinomyces viscosus	1	0.1
	Bacillus	4	0.41
	Finegoldia	4	0.41
	Finegoldia magna	4	0.41
	Kocuria	4	0.41
	Kocuria palustris	3	0.31
	Rhodococcus	4	0.41
	Haematobacter	3	0.31
	Haematobacter massiliensis	3	0.31
	Enterococcus	3	0.31
	Enterococcus faecalis	3	0.31
	Microbacterium	3	0.31
	Microbacterium oxydans	1	0.1
	Anaerococcus	2	0.2
	Anaerococcus prevotii	2	0.2
	Nocardiopsis	2	0.2
	Nocardiopsis dassonvillei	2	0.2
	Stenotrophomonas	1	0.1
	Stenotrophomonas maltophilia	1	0.1
	Aggregatibacter	1	0.1
	Aggregatibacter segnis	1	0.1
	Roseomonas	1	0.1
	Brevundimonas	1	0.1

Patient No.	Organism	Reads	RPM
	Neisseria	1	0.1
	Neisseria mucosa	1	0.1
	Campylobacter	1	0.1
	Campylobacter showae	1	0.1
	Fusobacterium	1	0.1
	Fusobacterium nucleatum	1	0.1
	Leptotrichia	1	0.1
	Leptotrichia wadei	1	0.1
	Prevotella	1	0.1
	Gemella	1	0.1
	Gemella morbillorum	1	0.1
	Exiguobacterium	1	0.1
	Rothia	1	0.1
	Cellulosimicrobium	1	0.1
	Aspergillus	73	7.42
	Aspergillus terreus	73	7.42
	Malassezia	13	1.32
	Malassezia restricta	11	1.12
	Malassezia globosa	2	0.2
	Nakaseomyces	3	0.31
	[Candida] glabrata	3	0.31
	Yarrowia	1	0.1
	Yarrowia lipolytica	1	0.1
7	Staphylococcus	989	115.94
	Staphylococcus aureus	968	113.48
	Staphylococcus hominis	2	0.23
	Staphylococcus epidermidis	1	0.12
	Cutibacterium	44	5.16
	Cutibacterium acnes	43	5.04
	Cutibacterium granulosum	1	0.12
	Pseudomonas	17	1.99
	Pseudomonas stutzeri	15	1.76
	Pseudomonas mendocina	1	0.12
	Nocardiopsis	13	1.52
	Nocardiopsis dassonvillei	12	1.41
	Streptomyces	11	1.29
	Bacillus	7	0.82
	Acinetobacter	6	0.7
	Acinetobacter johnsonii	5	0.59
	Stenotrophomonas	6	0.7
	Stenotrophomonas maltophilia	5	0.59
	Corynebacterium	6	0.7
	Corynebacterium segmentosum	3	0.35
	Corynebacterium simulans	1	0.12

Patient No.	Organism	Reads	RPM
	Corynebacterium stationis	1	0.12
	Streptococcus	3	0.35
	Streptococcus sanguinis	1	0.12
	Streptococcus parasanguinis	1	0.12
	Kocuria	3	0.35
	Kocuria palustris	3	0.35
	Moraxella	1	0.12
	Moraxella osloensis	1	0.12
	Achromobacter	1	0.12
	Achromobacter xylosoxidans	1	0.12
	Lautropia	1	0.12
	Lautropia mirabilis	1	0.12
	Lactobacillus	1	0.12
	Lactobacillus coryniformis	1	0.12
	Leuconostoc	1	0.12
	Micrococcus	1	0.12
	Aspergillus	58	6.8
	Aspergillus niger	9	1.06
	Aspergillus flavus	4	0.47
	Aspergillus fumigatus	1	0.12
	Aspergillus nidulans	1	0.12
	Alternaria	19	2.23
	Alternaria alternata	8	0.94
	Alternaria arborescens	4	0.47
	Malassezia	5	0.59
	Malassezia globosa	3	0.35
	Malassezia restricta	2	0.23
	Trichoderma	2	0.23
	Trichoderma harzianum	1	0.12
	Trichoderma asperellum	1	0.12
8	Proteus	48358	5969.3
	Proteus mirabilis	47590	5874.5
	Proteus vulgaris	56	6.91
	Proteus hauseri	30	3.7
	Prevotella	19821	2446.7
	Prevotella oris	17011	2099.83
	Prevotella intermedia	1845	227.75
	Prevotella enoeca	70	8.64
	Prevotella denticola	68	8.39
	Prevotella melaninogenica	63	7.78
	Prevotella scopos	18	2.22
	Prevotella fusca	12	1.48
	Prevotella dentalis	9	1.11
	Prevotella oulorum	2	0.25

Patient No.	Organism	Reads	RPM
	Prevotella stercorea	1	0.12
	Prevotella buccalis	1	0.12
	Parvimonas	8289	1023.19
	Parvimonas micra	8289	1023.19
	Campylobacter	7451	919.75
	Campylobacter showae	6869	847.91
	Campylobacter rectus	200	24.69
	Campylobacter gracilis	118	14.57
	Campylobacter concisus	82	10.12
	Campylobacter curvus	7	0.86
	Filifactor	5745	709.16
	Filifactor alocis	5745	709.16
	Cutibacterium	1914	236.26
	Cutibacterium acnes	1851	228.49
	Cutibacterium granulosum	48	5.93
	Cutibacterium avidum	1	0.12
	Mycoplasma	1863	229.97
	Mycoplasma salivarium	1856	229.1
	Peptoniphilus	1842	227.38
	Peptoniphilus rhinitidis	1248	154.05
	Peptoniphilus harei	572	70.61
	Mogibacterium	1682	207.63
	Mogibacterium diversum	1424	175.78
	Mogibacterium pumilum	204	25.18
	Pseudomonas	1184	146.15
	Pseudomonas stutzeri	1036	127.88
	Pseudomonas mendocina	36	4.44
	Pseudomonas aeruginosa	12	1.48
	Pseudomonas monteilii	4	0.49
	Pseudomonas sp. LG1D9	3	0.37
	Pseudomonas putida	3	0.37
	Pseudomonas oleovorans	2	0.25
	Pseudomonas oryzihabitans	2	0.25
	Pseudomonas fulva	1	0.12
	Pseudomonas protegens	1	0.12
	Fusobacterium	986	121.71
	Fusobacterium nucleatum	913	112.7
	Fusobacterium necrophorum	33	4.07
	Gemella	477	58.88
	Gemella morbillorum	465	57.4
	Gemella sanguinis	3	0.37
	Nocardiopsis	470	58.02
	Nocardiopsis dassonvillei	460	56.78
	Streptococcus	445	54.93

Patient No.	Organism	Reads	RPM
	Streptococcus anginosus	38	4.69
	Streptococcus pyogenes	29	3.58
	Streptococcus constellatus	27	3.33
	Streptococcus salivarius	26	3.21
	Streptococcus intermedius	14	1.73
	Streptococcus equi	12	1.48
	Streptococcus dysgalactiae	10	1.23
	Streptococcus agalactiae	8	0.99
	Streptococcus pseudoporcinus	4	0.49
	Streptococcus oralis	4	0.49
	Streptococcus gordonii	3	0.37
	Streptococcus sanguinis	3	0.37
	Streptococcus suis	3	0.37
	Streptococcus mutans	2	0.25
	Streptococcus mitis	2	0.25
	Streptococcus australis	1	0.12
	Eikenella	384	47.4
	Eikenella corrodens	381	47.03
	Stenotrophomonas	289	35.67
	Stenotrophomonas maltophilia	251	30.98
	Streptomyces	167	20.61
	Streptomyces albulus	1	0.12
	Porphyromonas	166	20.49
	Porphyromonas gingivalis	94	11.6
	Porphyromonas endodontalis	49	6.05
	Porphyromonas asaccharolytica	17	2.1
	Staphylococcus	152	18.76
	Staphylococcus epidermidis	57	7.04
	Staphylococcus capitis	34	4.2
	Staphylococcus hominis	15	1.85
	Staphylococcus cohnii	6	0.74
	Staphylococcus saprophyticus	5	0.62
	Staphylococcus haemolyticus	5	0.62
	Staphylococcus pasteuri	2	0.25
	Staphylococcus warneri	2	0.25
	Staphylococcus aureus	1	0.12
	Mammaliicoccus sciuri	1	0.12
	Staphylococcus lugdunensis	1	0.12
	Acinetobacter	151	18.64
	Acinetobacter baumannii	58	7.16
	Acinetobacter johnsonii	40	4.94
	Acinetobacter schindleri	8	0.99
	Acinetobacter lwoffii	6	0.74
	Acinetobacter pittii	4	0.49

Patient No.	Organism	Reads	RPM
	Acinetobacter ursingii	2	0.25
	Acinetobacter soli	2	0.25
	Acinetobacter junii	1	0.12
	Acinetobacter radioresistens	1	0.12
	Corynebacterium	149	18.39
	Corynebacterium striatum	30	3.7
	Corynebacterium simulans	25	3.09
	Corynebacterium segmentosum	10	1.23
	Corynebacterium jeikeium	6	0.74
	Corynebacterium diphtheriae	6	0.74
	Corynebacterium xerosis	4	0.49
	Corynebacterium aurimucosum	2	0.25
	Corynebacterium ureicelerivorans	1	0.12
	Corynebacterium kroppenstedtii	1	0.12
	Bacillus	100	12.34
	Bacillus licheniformis	19	2.35
	Bacillus subtilis	3	0.37
	Bacillus cereus group	2	0.25
	Bacillus thuringiensis	1	0.12
	Bacillus circulans	1	0.12
	Bacillus pumilus	1	0.12
	Burkholderia	97	11.97
	Burkholderia cepacia complex	88	10.86
	Burkholderia vietnamiensis	54	6.67
	Burkholderia contaminans	2	0.25
	Burkholderia pyrrocinia	1	0.12
	Burkholderia multivorans	1	0.12
	Burkholderia gladioli	7	0.86
	Sphingomonas	62	7.65
	Sphingomonas paucimobilis	2	0.25
	Tannerella	59	7.28
	Tannerella forsythia	59	7.28
	Micrococcus	40	4.94
	Micrococcus luteus	39	4.81
	Achromobacter	39	4.81
	Achromobacter xylosoxidans	30	3.7
	Achromobacter insolitus	1	0.12
	Moraxella	37	4.57
	Moraxella osloensis	37	4.57
	Kocuria	37	4.57
	Kocuria palustris	30	3.7
	Kocuria rosea	4	0.49
	Pseudonocardia	33	4.07
	Pseudonocardia autotrophica	2	0.25

Patient No.	Organism	Reads	RPM
	Anaerococcus	26	3.21
	Anaerococcus prevotii	7	0.86
	Bacteroides	24	2.96
	Bacteroides heparinolyticus	11	1.36
	Bacteroides zoogleoformans	6	0.74
	Bacteroides fragilis	4	0.49
	Brevibacterium	23	2.84
	Pantoea	22	2.72
	Pantoea dispersa	18	2.22
	Pantoea agglomerans	2	0.25
	Atopobium	19	2.35
	Lancefieldella parvula	19	2.35
	Neisseria	18	2.22
	Neisseria mucosa	7	0.86
	Neisseria subflava	3	0.37
	Neisseria flavescens	2	0.25
	Neisseria elongata	2	0.25
	Neisseria gonorrhoeae	1	0.12
	Neisseria meningitidis	1	0.12
	Treponema	18	2.22
	Treponema putidum	6	0.74
	Treponema denticola	1	0.12
	Enterobacter	16	1.98
	Enterobacter cloacae complex	12	1.48
	Enterobacter cloacae	8	0.99
	Enterobacter ludwigii	1	0.12
	Gordonia	16	1.98
	Gordonia rubripertincta	1	0.12
	Methylobacterium	13	1.6
	Methylobacterium mesophilicum	1	0.12
	Paracoccus	13	1.6
	Paracoccus yeei	5	0.62
	Klebsiella	12	1.48
	Klebsiella pneumoniae	12	1.48
	Actinomyces	12	1.48
	Actinomyces oris	4	0.49
	Pannonibacter	10	1.23
	Pannonibacter phragmitetus	10	1.23
	Pluralibacter	9	1.11
	Pluralibacter gergoviae	9	1.11
	Rhizobium	9	1.11
	Brevundimonas	9	1.11
	Brevundimonas vesicularis	1	0.12
	Dietzia	8	0.99

Patient No.	Organism	Reads	RPM
	Chryseobacterium	7	0.86
	Chryseobacterium gallinarum	1	0.12
	Lactobacillus	7	0.86
	Lactiplantibacillus plantarum	1	0.12
	Xanthomonas	6	0.74
	Xanthomonas campestris	3	0.37
	Janibacter	6	0.74
	Janibacter melonis	4	0.49
	Janibacter indicus	1	0.12
	Acetobacter	5	0.62
	Exiguobacterium	5	0.62
	Exiguobacterium sp. AT1b	4	0.49
	Veillonella	5	0.62
	Veillonella parvula	5	0.62
	Mobiluncus	5	0.62
	Mobiluncus curtisii	5	0.62
	Dermabacter	5	0.62
	Microbacterium	5	0.62
	Microbacterium testaceum	1	0.12
	Providencia	4	0.49
	Providencia rettgeri	4	0.49
	Enterococcus	4	0.49
	Enterococcus faecalis	4	0.49
	Bifidobacterium	4	0.49
	Bifidobacterium longum	4	0.49
	Tsukamurella	4	0.49
	Tsukamurella tyrosinosolvens	4	0.49
	Aeromonas	3	0.37
	Haemophilus	3	0.37
	Haemophilus parainfluenzae	2	0.25
	[Haemophilus] ducreyi	1	0.12
	Lautropia	3	0.37
	Lautropia mirabilis	3	0.37
	Paraburkholderia	3	0.37
	Paraburkholderia tropica	1	0.12
	Blautia	3	0.37
	[Ruminococcus] gnavus	1	0.12
	Clostridioides	3	0.37
	Clostridioides difficile	3	0.37
	Finegoldia	3	0.37
	Finegoldia magna	3	0.37
	Kytococcus	3	0.37
	Kytococcus sedentarius	3	0.37
	Rhodococcus	3	0.37

Patient No.	Organism	Reads	RPM
	Agrobacterium	2	0.25
	Agrobacterium tumefaciens	1	0.12
	Methylorubrum	2	0.25
	Methylorubrum populi	2	0.25
	Roseomonas	2	0.25
	Janthinobacterium	2	0.25
	Comamonas	2	0.25
	Comamonas testosteroni	1	0.12
	Delftia	2	0.25
	Delftia tsuruhatensis	2	0.25
	Dialister	2	0.25
	Dialister pneumosintes	1	0.12
	Gardnerella	2	0.25
	Gardnerella vaginalis	2	0.25
	Rothia	2	0.25
	Rothia mucilaginosa	1	0.12
	Rothia dentocariosa	1	0.12
	Curtobacterium	2	0.25
	Dermacoccus	2	0.25
	Dermacoccus nishinomiyaensis	2	0.25
	Mycolicibacterium	2	0.25
	Mycolicibacterium smegmatis	2	0.25
	Olsenella	2	0.25
	Olsenella uli	1	0.12
	Serratia	1	0.12
	Serratia marcescens	1	0.12
	Legionella	1	0.12
	Legionella pneumophila	1	0.12
	Cardiobacterium	1	0.12
	Cardiobacterium hominis	1	0.12
	Aggregatibacter	1	0.12
	Aggregatibacter aphrophilus	1	0.12
	Aureimonas	1	0.12
	Aureimonas altamirensis	1	0.12
	Haematobacter	1	0.12
	Haematobacter massiliensis	1	0.12
	Ralstonia	1	0.12
	Ralstonia pickettii	1	0.12
	Lactococcus	1	0.12
	Lactococcus lactis	1	0.12
	Leuconostoc	1	0.12
	Leuconostoc mesenteroides	1	0.12
	Aerococcus	1	0.12
	Helcococcus	1	0.12

Patient No.	Organism	Reads	RPM
	Helcococcus kunzii	1	0.12
	Saccharomonospora	1	0.12
	Saccharomonospora viridis	1	0.12
	Aspergillus	2123	262.06
	Aspergillus niger	262	32.34
	Aspergillus nidulans	151	18.64
	Aspergillus flavus	130	16.05
	Aspergillus terreus	31	3.83
	Aspergillus glaucus	30	3.7
	Aspergillus oryzae	25	3.09
	Aspergillus nomius	6	0.74
	Aspergillus fumigatus	3	0.37
	Aspergillus versicolor	2	0.25
	Alternaria	395	48.76
	Alternaria alternata	248	30.61
	Alternaria arborescens	30	3.7
	Talaromyces	297	36.66
	Talaromyces pinophilus	16	1.98
	Talaromyces atroroseus	1	0.12
	Malassezia	149	18.39
	Malassezia restricta	120	14.81
	Malassezia globosa	29	3.58
	Trichoderma	138	17.03
	Trichoderma harzianum	101	12.47
	Trichoderma asperellum	11	1.36
	Trichoderma virens	5	0.62
	Trichoderma atroviride	4	0.49
	Trichoderma reesei	3	0.37
	Trichoderma gamsii	1	0.12
	Fusarium	17	2.1
	Fusarium verticillioides	5	0.62
	Fusarium fujikuroi	3	0.37
	Fusarium proliferatum	3	0.37
	Fusarium oxysporum	1	0.12
	Fusarium culmorum	1	0.12
	Candida	16	1.98
	Candida parapsilosis	16	1.98
	Lichtheimia	11	1.36
	Lichtheimia ramosa	11	1.36
	Penicillium	10	1.23
	Penicillium rubens	10	1.23
	Meyerozyma	5	0.62
	Meyerozyma guilliermondii	5	0.62
	Phaeoacremonium	5	0.62

Patient No.	Organism	Reads	RPM
	Phaeoacremonium minimum	5	0.62
	Yarrowia	4	0.49
	Yarrowia lipolytica	4	0.49
	Chaetomium	4	0.49
	Chaetomium globosum	4	0.49
	Debaryomyces	3	0.37
	Debaryomyces fabryi	2	0.25
	Debaryomyces hansenii	1	0.12
	Curvularia	2	0.25
	Curvularia lunata	1	0.12
	Schizophyllum	2	0.25
	Schizophyllum commune	2	0.25
	Wallemia	2	0.25
	Wallemia mellicola	2	0.25
	Exophiala	1	0.12
	Exophiala dermatitidis	1	0.12
	Trichomonas	12	1.48
	Trichomonas vaginalis	12	1.48
9	Streptomyces	277	37.83
	Pseudomonas	259	35.37
	Pseudomonas stutzeri	218	29.77
	Pseudomonas mendocina	16	2.19
	Pseudomonas aeruginosa	2	0.27
	Pseudomonas oryzihabitans	1	0.14
	Pseudomonas putida	1	0.14
	Pseudomonas entomophila	1	0.14
	Nocardiopsis	87	11.88
	Nocardiopsis dassonvillei	83	11.34
	Stenotrophomonas	46	6.28
	Stenotrophomonas maltophilia	40	5.46
	Pseudonocardia	40	5.46
	Pseudonocardia autotrophica	2	0.27
	Cutibacterium	36	4.92
	Cutibacterium acnes	34	4.64
	Cutibacterium granulosum	2	0.27
	Bacillus	33	4.51
	Bacillus circulans	2	0.27
	Moraxella	29	3.96
	Moraxella osloensis	29	3.96
	Acinetobacter	22	3
	Acinetobacter johnsonii	9	1.23
	Acinetobacter schindleri	5	0.68
	Acinetobacter junii	1	0.14
	Acinetobacter lwoffii	1	0.14

Patient No.	Organism	Reads	RPM
	Achromobacter	18	2.46
	Achromobacter xylosoxidans	16	2.19
	Staphylococcus	17	2.32
	Staphylococcus epidermidis	5	0.68
	Staphylococcus cohnii	2	0.27
	Staphylococcus aureus	2	0.27
	Staphylococcus pettenkoferi	1	0.14
	Staphylococcus haemolyticus	1	0.14
	Staphylococcus hominis	1	0.14
	Staphylococcus pasteuri	1	0.14
	Burkholderia	16	2.19
	Burkholderia cepacia complex	6	0.82
	Burkholderia vietnamiensis	4	0.55
	Burkholderia contaminans	2	0.27
	Burkholderia gladioli	3	0.41
	Sphingomonas	15	2.05
	Sphingomonas koreensis	1	0.14
	Kocuria	11	1.5
	Kocuria palustris	10	1.37
	Pluralibacter	6	0.82
	Pluralibacter gergoviae	6	0.82
	Paracoccus	5	0.68
	Paracoccus yeei	1	0.14
	Actinomyces	5	0.68
	Actinomyces naeslundii	1	0.14
	Pauljensenia hongkongensis	1	0.14
	Microbacterium	5	0.68
	Microbacterium foliorum	1	0.14
	Corynebacterium	5	0.68
	Corynebacterium matruchotii	1	0.14
	Corynebacterium segmentosum	1	0.14
	Exiguobacterium	4	0.55
	Exiguobacterium sp. AT1b	3	0.41
	Micrococcus	4	0.55
	Micrococcus luteus	4	0.55
	Pantoea	3	0.41
	Pantoea dispersa	3	0.41
	Methylobacterium	3	0.41
	Methylorubrum	3	0.41
	Gordonia	3	0.41
	Gordonia terrae	1	0.14
	Aeromonas	2	0.27
	Aeromonas caviae	1	0.14
	Roseomonas	2	0.27

Patient No.	Organism	Reads	RPM
	Pannonibacter	2	0.27
	Pannonibacter phragmitetus	2	0.27
	Comamonas	2	0.27
	Comamonas terrigena	1	0.14
	Streptococcus	2	0.27
	Streptococcus thermophilus	1	0.14
	Lactobacillus	2	0.27
	Lactobacillus iners	1	0.14
	Finegoldia	2	0.27
	Finegoldia magna	2	0.27
	Janibacter	2	0.27
	Janibacter indicus	2	0.27
	Enterobacter	1	0.14
	Klebsiella	1	0.14
	Cronobacter	1	0.14
	Xanthomonas	1	0.14
	Xanthomonas campestris	1	0.14
	Prevotella	1	0.14
	Prevotella melaninogenica	1	0.14
	Macrococcus	1	0.14
	Macrococcus caseolyticus	1	0.14
	Enterococcus	1	0.14
	Enterococcus avium	1	0.14
	Kytococcus	1	0.14
	Kytococcus sedentarius	1	0.14
	Aspergillus	452	61.73
	Aspergillus niger	42	5.74
	Aspergillus flavus	38	5.19
	Aspergillus oryzae	9	1.23
	Aspergillus nidulans	6	0.82
	Aspergillus fumigatus	5	0.68
	Aspergillus terreus	5	0.68
	Aspergillus glaucus	4	0.55
	Aspergillus bombycis	1	0.14
	Alternaria	116	15.84
	Alternaria alternata	66	9.01
	Alternaria arborescens	7	0.96
	Talaromyces	44	6.01
	Talaromyces pinophilus	4	0.55
	Talaromyces atroroseus	1	0.14
	Trichoderma	23	3.14
	Trichoderma harzianum	17	2.32
	Trichoderma asperellum	3	0.41
	Trichoderma reesei	1	0.14

Patient No.	Organism	Reads	RPM
	Penicillium	5	0.68
	Penicillium rubens	4	0.55
	Penicillium digitatum	1	0.14
	Debaryomyces	4	0.55
	Debaryomyces hansenii	4	0.55
	Phaeoacremonium	4	0.55
	Phaeoacremonium minimum	4	0.55
	Meyerozyma	3	0.41
	Meyerozyma guilliermondii	3	0.41
	Malassezia	3	0.41
	Malassezia restricta	3	0.41
	Yarrowia	2	0.27
	Yarrowia lipolytica	2	0.27
	Fusarium	2	0.27
	Fusarium culmorum	1	0.14
	Fusarium verticillioides	1	0.14
	unclassified Anelloviridae	1	0.14
	Torque teno virus	1	0.14
10	Corynebacterium	4541295	301068
	Corynebacterium segmentosum	4007528	265681.6
	Corynebacterium macginleyi	99230	6578.51
	Corynebacterium striatum	40547	2688.09
	Corynebacterium simulans	17876	1185.1
	Corynebacterium aurimucosum	15166	1005.44
	Corynebacterium diphtheriae	12697	841.76
	Corynebacterium pseudodiphtheriticum	11269	747.09
	Corynebacterium accolens	6783	449.68
	Corynebacterium propinquum	3867	256.37
	Corynebacterium camporealensis	2624	173.96
	Corynebacterium resistens	2039	135.18
	Corynebacterium timonense	1801	119.4
	Corynebacterium jeikeium	1371	90.89
	Corynebacterium ureicelerivorans	1283	85.06
	Corynebacterium afermentans	678	44.95
	Corynebacterium tuscaniense	429	28.44
	Corynebacterium tuberculostearicum	426	28.24
	Corynebacterium urealyticum	353	23.4
	Corynebacterium humireducens	301	19.95
	Corynebacterium stationis	151	10.01
	Corynebacterium halotolerans	148	9.81
	Corynebacterium renale	107	7.09
	Corynebacterium falsenii	99	6.56
	Corynebacterium otitidis	95	6.3
	Corynebacterium riegelii	81	5.37

Patient No.	Organism	Reads	RPM
	Corynebacterium xerosis	31	2.06
	Corynebacterium minutissimum	24	1.59
	Corynebacterium glucuronolyticum	18	1.19
	Corynebacterium kroppenstedtii	17	1.13
	Corynebacterium argentoratense	16	1.06
	Corynebacterium matruchotii	15	0.99
	Corynebacterium pseudotuberculosis	15	0.99
	Corynebacterium ulcerans	13	0.86
	Corynebacterium durum	1	0.07
	Corynebacterium confusum	1	0.07
	Staphylococcus	241076	15982.28
	Staphylococcus lugdunensis	142283	9432.74
	Staphylococcus caprae	80315	5324.53
	Staphylococcus pettenkoferi	8446	559.93
	Staphylococcus aureus	2267	150.29
	Staphylococcus epidermidis	889	58.94
	Staphylococcus capitis	102	6.76
	Staphylococcus nepalensis	95	6.3
	Staphylococcus auricularis	94	6.23
	Staphylococcus hominis	79	5.24
	Staphylococcus saprophyticus	41	2.72
	Staphylococcus haemolyticus	36	2.39
	Mammaliicoccus stepanovicii	29	1.92
	Staphylococcus simulans	12	0.8
	Staphylococcus pasteuri	7	0.46
	Staphylococcus pseudintermedius	7	0.46
	Staphylococcus warneri	7	0.46
	Staphylococcus equorum	6	0.4
	Mammaliicoccus sciuri	6	0.4
	Staphylococcus cohnii	5	0.33
	Staphylococcus arlettae	3	0.2
	Staphylococcus agnetis	2	0.13
	Staphylococcus kloosii	2	0.13
	Staphylococcus xylosus	2	0.13
	Staphylococcus condimenti	1	0.07
	Staphylococcus felis	1	0.07
	Staphylococcus simiae	1	0.07
	Pseudomonas	2708	179.53
	Pseudomonas stutzeri	2185	144.86
	Pseudomonas mendocina	153	10.14
	Pseudomonas aeruginosa	69	4.57
	Pseudomonas oleovorans	13	0.86
	Pseudomonas putida	11	0.73
	Pseudomonas monteilii	9	0.6

Patient No.	Organism	Reads	RPM
	Pseudomonas fluorescens	7	0.46
	Pseudomonas luteola	4	0.27
	Pseudomonas fulva	3	0.2
	Pseudomonas oryzihabitans	3	0.2
	Pseudomonas sp. LG1D9	1	0.07
	Pseudomonas protegens	1	0.07
	Streptomyces	1935	128.28
	Streptomyces cattleya	2	0.13
	Streptomyces albulus	2	0.13
	Streptomyces atratus	1	0.07
	Nocardiopsis	861	57.08
	Nocardiopsis dassonvillei	823	54.56
	Stenotrophomonas	700	46.41
	Stenotrophomonas maltophilia	673	44.62
	Stenotrophomonas acidaminiphila	8	0.53
	Haemophilus	424	28.11
	[Haemophilus] ducreyi	418	27.71
	Haemophilus parainfluenzae	3	0.2
	Haemophilus haemolyticus	1	0.07
	Haemophilus parahaemolyticus	1	0.07
	Brevibacterium	329	21.81
	Bacillus	325	21.55
	Bacillus licheniformis	83	5.5
	Bacillus subtilis	17	1.13
	Bacillus circulans	6	0.4
	Bacillus cereus group	1	0.07
	Bacillus glycinifermentans	1	0.07
	Priestia megaterium	1	0.07
	Bacillus pumilus	1	0.07
	Acinetobacter	280	18.56
	Acinetobacter johnsonii	107	7.09
	Acinetobacter schindleri	30	1.99
	Acinetobacter soli	21	1.39
	Acinetobacter lwoffii	19	1.26
	Acinetobacter baumannii	12	0.8
	Acinetobacter junii	6	0.4
	Acinetobacter ursingii	3	0.2
	Acinetobacter haemolyticus	3	0.2
	Acinetobacter radioresistens	2	0.13
	Acinetobacter gyllenbergii	2	0.13
	Acinetobacter nosocomialis	1	0.07
	Acinetobacter pittii	1	0.07
	Paenibacillus	251	16.64
	Pseudonocardia	229	15.18

Patient No.	Organism	Reads	RPM
	Pseudonocardia autotrophica	15	0.99
	Pantoea	226	14.98
	Pantoea dispersa	212	14.05
	Pantoea agglomerans	9	0.6
	Prevotella	179	11.87
	Prevotella intermedia	59	3.91
	Prevotella nigrescens	35	2.32
	Prevotella melaninogenica	19	1.26
	Prevotella buccae	15	0.99
	Prevotella oralis	13	0.86
	Prevotella scopos	6	0.4
	Prevotella oris	4	0.27
	Prevotella fusca	4	0.27
	Prevotella enoeca	2	0.13
	Prevotella denticola	2	0.13
	Prevotella jejuni	2	0.13
	Achromobacter	174	11.54
	Achromobacter xylosoxidans	96	6.36
	Achromobacter denitrificans	6	0.4
	Achromobacter insolitus	2	0.13
	Moraxella	138	9.15
	Moraxella osloensis	138	9.15
	Burkholderia	132	8.75
	Burkholderia cepacia complex	127	8.42
	Burkholderia vietnamiensis	61	4.04
	Burkholderia contaminans	23	1.52
	Burkholderia pyrrocinia	1	0.07
	Burkholderia multivorans	1	0.07
	Burkholderia gladioli	2	0.13
	Streptococcus	114	7.56
	Streptococcus anginosus	13	0.86
	Streptococcus gordonii	9	0.6
	Streptococcus mitis	6	0.4
	Streptococcus oralis	5	0.33
	Streptococcus australis	4	0.27
	Streptococcus pasteurianus	4	0.27
	Streptococcus pneumoniae	4	0.27
	Streptococcus parasanguinis	4	0.27
	Streptococcus cristatus	3	0.2
	Streptococcus salivarius	1	0.07
	Streptococcus sanguinis	1	0.07
	Streptococcus thermophilus	1	0.07
	Streptococcus intermedius	1	0.07
	Streptococcus vestibularis	1	0.07

Patient No.	Organism	Reads	RPM
	Micrococcus	98	6.5
	Micrococcus luteus	92	6.1
	Amycolatopsis	96	6.36
	Lactobacillus	95	6.3
	Lactiplantibacillus plantarum	81	5.37
	Lactobacillus crispatus	3	0.2
	Lactobacillus iners	1	0.07
	Bordetella	76	5.04
	Bordetella petrii	2	0.13
	Bordetella hinzii	1	0.07
	Dermabacter	75	4.97
	Dermabacter hominis	1	0.07
	Massilia	70	4.64
	Massilia timonae	36	2.39
	Methylobacterium	69	4.57
	Methylobacterium aquaticum	3	0.2
	Methylobacterium mesophilicum	1	0.07
	Methylobacterium radiotolerans	1	0.07
	Kocuria	69	4.57
	Kocuria palustris	53	3.51
	Kocuria rosea	8	0.53
	Kocuria rhizophila	2	0.13
	Porphyromonas	54	3.58
	Porphyromonas gingivalis	53	3.51
	Ochrobactrum	43	2.85
	Brucella intermedia	32	2.12
	Ochrobactrum anthropi	10	0.66
	Pannonibacter	43	2.85
	Pannonibacter phragmitetus	43	2.85
	Lactococcus	37	2.45
	Lactococcus lactis	37	2.45
	Roseomonas	35	2.32
	Roseomonas mucosa	33	2.19
	Saccharopolyspora	34	2.25
	Saccharopolyspora rectivirgula	27	1.79
	Actinomyces	33	2.19
	Actinomyces naeslundii	5	0.33
	Actinomyces oris	3	0.2
	Actinomyces viscosus	2	0.13
	Fusobacterium	32	2.12
	Fusobacterium nucleatum	28	1.86
	Veillonella	29	1.92
	Veillonella parvula	24	1.59
	Veillonella dispar	2	0.13

Patient No.	Organism	Reads	RPM
	Serratia	27	1.79
	Serratia rubidaea	27	1.79
	Brevundimonas	27	1.79
	Brevundimonas vesicularis	3	0.2
	Rothia	27	1.79
	Rothia dentocariosa	11	0.73
	Rothia mucilaginosa	10	0.66
	Rhodococcus	23	1.52
	Rhodococcus fascians	3	0.2
	Rhodococcus erythropolis	2	0.13
	Rhodococcus hoagii	1	0.07
	Rhodococcus rhodochrous	1	0.07
	Janibacter	21	1.39
	Janibacter melonis	10	0.66
	Janibacter indicus	4	0.27
	Enterobacter	20	1.33
	Enterobacter cloacae complex	16	1.06
	Enterobacter cloacae	5	0.33
	Sphingomonas	20	1.33
	Sphingomonas koreensis	7	0.46
	Sphingomonas paucimobilis	5	0.33
	Dietzia	19	1.26
	Dietzia natronolimnaea	10	0.66
	Exiguobacterium	18	1.19
	Exiguobacterium sp. AT1b	17	1.13
	Methylorubrum	17	1.13
	Methylorubrum populi	12	0.8
	Microbacterium	17	1.13
	Microbacterium paraoxydans	2	0.13
	Microbacterium oxydans	1	0.07
	Microbacterium foliorum	1	0.07
	Xanthomonas	14	0.93
	Xanthomonas campestris	5	0.33
	Bifidobacterium	14	0.93
	Bifidobacterium dentium	14	0.93
	Leuconostoc	13	0.86
	Leuconostoc pseudomesenteroides	9	0.6
	Leuconostoc citreum	1	0.07
	Leuconostoc mesenteroides	1	0.07
	Cutibacterium	13	0.86
	Cutibacterium granulosum	7	0.46
	Cutibacterium acnes	3	0.2
	Cutibacterium avidum	1	0.07
	Campylobacter	12	0.8

Patient No.	Organism	Reads	RPM
	Campylobacter concisus	11	0.73
	Campylobacter showae	1	0.07
	Cellulosimicrobium	12	0.8
	Cellulosimicrobium cellulans	12	0.8
	Agrobacterium	11	0.73
	Agrobacterium tumefaciens	9	0.6
	Comamonas	11	0.73
	Comamonas kerstersii	2	0.13
	Comamonas testosteroni	1	0.07
	Comamonas terrigena	1	0.07
	Gordonia	11	0.73
	Gordonia terrae	2	0.13
	Neisseria	10	0.66
	Neisseria subflava	2	0.13
	Neisseria flavescens	1	0.07
	Neisseria elongata	1	0.07
	Tannerella	10	0.66
	Tannerella forsythia	10	0.66
	Dermacoccus	10	0.66
	Dermacoccus nishinomiyaensis	9	0.6
	Nocardia	10	0.66
	Nocardia farcinica	6	0.4
	Nocardia cyriacigeorgica	3	0.2
	Klebsiella	9	0.6
	Klebsiella aerogenes	1	0.07
	Klebsiella oxytoca	1	0.07
	Paracoccus	9	0.6
	Paracoccus yeei	6	0.4
	Kytococcus	9	0.6
	Kytococcus sedentarius	9	0.6
	Ralstonia	8	0.53
	Ralstonia insidiosa	3	0.2
	Ralstonia pickettii	2	0.13
	Aeromonas	7	0.46
	Aeromonas caviae	3	0.2
	Aeromonas salmonicida	1	0.07
	Weissella	7	0.46
	Weissella confusa	2	0.13
	Mycolicibacterium	7	0.46
	Mycolicibacterium smegmatis	6	0.4
	Mycolicibacterium fortuitum	1	0.07
	Chryseobacterium	6	0.4
	Chryseobacterium gleum	1	0.07
	Capnocytophaga	5	0.33

Patient No.	Organism	Reads	RPM
	Capnocytophaga sputigena	2	0.13
	Capnocytophaga gingivalis	1	0.07
	Capnocytophaga leadbetteri	1	0.07
	Pluralibacter	4	0.27
	Pluralibacter gergoviae	4	0.27
	Leptotrichia	4	0.27
	Leptotrichia wadei	2	0.13
	Leptotrichia buccalis	1	0.07
	Isoptericola	4	0.27
	Isoptericola variabilis	3	0.2
	Mycobacteroides	4	0.27
	Mycobacteroides saopaulense	4	0.27
	Citrobacter	3	0.2
	Citrobacter koseri	1	0.07
	Legionella	3	0.2
	Legionella pneumophila	3	0.2
	Azospirillum	3	0.2
	Azospirillum brasilense	1	0.07
	Delftia	3	0.2
	Delftia tsuruhatensis	1	0.07
	Lautropia	3	0.2
	Lautropia mirabilis	3	0.2
	Cupriavidus	3	0.2
	Cupriavidus gilardii	2	0.13
	Cupriavidus pauculus	1	0.07
	Empedobacter	3	0.2
	Empedobacter brevis	3	0.2
	Enterococcus	3	0.2
	Enterococcus casseliflavus	2	0.13
	Enterococcus gallinarum	1	0.07
	Psychrobacter	2	0.13
	Psychrobacter cryohalolentis	2	0.13
	Providencia	2	0.13
	Providencia rettgeri	2	0.13
	Shewanella	2	0.13
	Shewanella putrefaciens	2	0.13
	Vibrio	2	0.13
	Vibrio cholerae	1	0.07
	Aggregatibacter	2	0.13
	Aggregatibacter segnis	2	0.13
	Flavobacterium	2	0.13
	Flavobacterium psychrophilum	2	0.13
	Gemella	2	0.13
	Gemella sanguinis	1	0.07

Patient No.	Organism	Reads	RPM
	Gemella haemolysans	1	0.07
	Jonesia	2	0.13
	Jonesia denitrificans	2	0.13
	Mycobacterium	2	0.13
	Mycobacterium kansasii	1	0.07
	Mycobacterium marinum	1	0.07
	Leclercia	1	0.07
	Leclercia adecarboxylata	1	0.07
	Cronobacter	1	0.07
	Lelliottia	1	0.07
	Lelliottia amnigena	1	0.07
	Rickettsia	1	0.07
	Rickettsia felis	1	0.07
	Haematobacter	1	0.07
	Haematobacter massiliensis	1	0.07
	Eikenella	1	0.07
	Eikenella corrodens	1	0.07
	Arcobacter	1	0.07
	Arcobacter cryaerophilus	1	0.07
	Treponema	1	0.07
	Treponema denticola	1	0.07
	Lysinibacillus	1	0.07
	Dolosigranulum	1	0.07
	Dolosigranulum pigrum	1	0.07
	Clostridium	1	0.07
	Clostridium butyricum	1	0.07
	Aspergillus	8847	586.52
	Aspergillus niger	1159	76.84
	Aspergillus flavus	678	44.95
	Aspergillus glaucus	186	12.33
	Aspergillus nidulans	141	9.35
	Aspergillus oryzae	141	9.35
	Aspergillus fumigatus	37	2.45
	Aspergillus terreus	25	1.66
	Aspergillus nomius	14	0.93
	Aspergillus bombycis	6	0.4
	Aspergillus ruber	3	0.2
	Aspergillus versicolor	3	0.2
	Aspergillus candidus	1	0.07
	Aspergillus campestris	1	0.07
	Aspergillus fischeri	1	0.07
	Alternaria	2925	193.91
	Alternaria alternata	1713	113.56
	Alternaria arborescens	299	19.82

Patient No.	Organism	Reads	RPM
	Talaromyces	823	54.56
	Talaromyces pinophilus	81	5.37
	Talaromyces atroroseus	12	0.8
	Talaromyces marneffei	1	0.07
	Trichoderma	514	34.08
	Trichoderma harzianum	340	22.54
	Trichoderma virens	37	2.45
	Trichoderma asperellum	34	2.25
	Trichoderma atroviride	27	1.79
	Trichoderma reesei	13	0.86
	Trichoderma gamsii	13	0.86
	Malassezia	84	5.57
	Malassezia restricta	80	5.3
	Malassezia slooffiae	3	0.2
	Malassezia globosa	1	0.07
	Penicillium	65	4.31
	Penicillium rubens	64	4.24
	Fusarium	54	3.58
	Fusarium fujikuroi	15	0.99
	Fusarium verticillioides	15	0.99
	Fusarium incarnatum-equiseti species complex	8	0.53
	Fusarium proliferatum	3	0.2
	Fusarium oxysporum	1	0.07
	Fusarium solani species complex	1	0.07
	Meyerozyma	18	1.19
	Meyerozyma guilliermondii	18	1.19
	Phaeoacremonium	12	0.8
	Phaeoacremonium minimum	12	0.8
	Lichtheimia	9	0.6
	Lichtheimia ramosa	9	0.6
	Yarrowia	8	0.53
	Yarrowia lipolytica	8	0.53
	Chaetomium	7	0.46
	Chaetomium globosum	7	0.46
	Candida	6	0.4
	Candida parapsilosis	3	0.2
	Candida tropicalis	3	0.2
	Schizophyllum	5	0.33
	Schizophyllum commune	5	0.33
	Saccharomyces	3	0.2
	Saccharomyces cerevisiae	3	0.2
	Exophiala	3	0.2
	Exophiala oligosperma	2	0.13
	Exophiala xenobiotica	1	0.07

Patient No.	Organism	Reads	RPM
	Curvularia	3	0.2
	Curvularia lunata	1	0.07
	Debaryomyces	2	0.13
	Debaryomyces fabryi	1	0.07
	Debaryomyces hansenii	1	0.07
	Pichia	2	0.13
	Pichia kudriavzevii	2	0.13
	Syncephalastrum	1	0.07
	Syncephalastrum monosporum	1	0.07
	Purpureocillium	1	0.07
	Purpureocillium lilacinum	1	0.07
	Trichosporon	1	0.07
	Trichosporon asahii	1	0.07
	Mastadenovirus	1	0.07
11	Staphylococcus	1435881	116861.9
	Staphylococcus epidermidis	1285693	104638.5
	Staphylococcus lugdunensis	93808	7634.74
	Staphylococcus aureus	4786	389.52
	Staphylococcus hominis	2798	227.72
	Staphylococcus capitis	2743	223.24
	Staphylococcus haemolyticus	237	19.29
	Staphylococcus caprae	153	12.45
	Staphylococcus nepalensis	52	4.23
	Staphylococcus warneri	43	3.5
	Staphylococcus pettenkoferi	23	1.87
	Staphylococcus equorum	19	1.55
	Staphylococcus simulans	19	1.55
	Staphylococcus arlettae	18	1.46
	Staphylococcus xylosus	16	1.3
	Staphylococcus cohnii	15	1.22
	Staphylococcus saprophyticus	14	1.14
	Staphylococcus gallinarum	14	1.14
	Staphylococcus saccharolyticus	3	0.24
	Staphylococcus chromogenes	1	0.08
	Staphylococcus kloosii	1	0.08
	Staphylococcus argenteus	1	0.08
	Pseudomonas	504	41.02
	Pseudomonas stutzeri	441	35.89
	Pseudomonas mendocina	19	1.55
	Pseudomonas aeruginosa	4	0.33
	Pseudomonas fluorescens	3	0.24
	Pseudomonas putida	2	0.16
	Pseudomonas alcaligenes	1	0.08
	Pseudomonas luteola	1	0.08

Patient No.	Organism	Reads	RPM
	Pseudomonas oleovorans	1	0.08
	Streptomyces	120	9.77
	Streptomyces atratus	1	0.08
	Nocardiopsis	113	9.2
	Nocardiopsis dassonvillei	112	9.12
	Acinetobacter	63	5.13
	Acinetobacter johnsonii	23	1.87
	Acinetobacter lwoffii	4	0.33
	Acinetobacter junii	2	0.16
	Acinetobacter soli	1	0.08
	Acinetobacter ursingii	1	0.08
	Acinetobacter schindleri	1	0.08
	Stenotrophomonas	60	4.88
	Stenotrophomonas maltophilia	59	4.8
	Pseudonocardia	58	4.72
	Pseudonocardia autotrophica	3	0.24
	Corynebacterium	54	4.39
	Corynebacterium segmentosum	32	2.6
	Corynebacterium kroppenstedtii	2	0.16
	Corynebacterium matruchotii	2	0.16
	Corynebacterium falsenii	2	0.16
	Corynebacterium ureicelerivorans	1	0.08
	Corynebacterium resistens	1	0.08
	Corynebacterium diphtheriae	1	0.08
	Moraxella	34	2.77
	Moraxella osloensis	34	2.77
	Burkholderia	30	2.44
	Burkholderia cepacia complex	30	2.44
	Burkholderia vietnamiensis	11	0.9
	Burkholderia contaminans	7	0.57
	Burkholderia multivorans	2	0.16
	Burkholderia cenocepacia	1	0.08
	Micrococcus	28	2.28
	Micrococcus luteus	27	2.2
	Kocuria	26	2.12
	Kocuria palustris	8	0.65
	Kocuria rosea	7	0.57
	Kocuria rhizophila	4	0.33
	Ochrobactrum	21	1.71
	Brucella intermedia	21	1.71
	Streptococcus	20	1.63
	Streptococcus oralis	6	0.49
	Streptococcus gordonii	4	0.33
	Streptococcus sanguinis	3	0.24

Patient No.	Organism	Reads	RPM
	Streptococcus mitis	2	0.16
	Streptococcus australis	1	0.08
	Methylobacterium	17	1.38
	Methylobacterium radiotolerans	5	0.41
	Methylobacterium aquaticum	1	0.08
	Achromobacter	17	1.38
	Achromobacter xylosoxidans	16	1.3
	Achromobacter denitrificans	1	0.08
	Cutibacterium	15	1.22
	Cutibacterium granulosum	14	1.14
	Cutibacterium acnes	1	0.08
	Pluralibacter	12	0.98
	Pluralibacter gergoviae	12	0.98
	Bacillus	11	0.9
	Bacillus licheniformis	2	0.16
	Bacillus subtilis	2	0.16
	Bacillus cereus group	1	0.08
	Priestia megaterium	1	0.08
	Brevundimonas	9	0.73
	Brevundimonas vesicularis	1	0.08
	Brevibacterium	8	0.65
	Janibacter	8	0.65
	Janibacter indicus	6	0.49
	Janibacter melonis	1	0.08
	Sphingomonas	7	0.57
	Sphingomonas paucimobilis	1	0.08
	Actinomyces	7	0.57
	Actinomyces oris	2	0.16
	Actinomyces naeslundii	1	0.08
	Methylorubrum	5	0.41
	Methylorubrum populi	5	0.41
	Rothia	5	0.41
	Rothia mucilaginosa	2	0.16
	Rothia aeria	1	0.08
	Rothia dentocariosa	1	0.08
	Alcaligenes	4	0.33
	Alcaligenes faecalis	4	0.33
	Lactobacillus	4	0.33
	Lacticaseibacillus paracasei	1	0.08
	Lactobacillus iners	1	0.08
	Leuconostoc	4	0.33
	Leuconostoc pseudomesenteroides	1	0.08
	Dermacoccus	4	0.33
	Dermacoccus nishinomiyaensis	4	0.33

Patient No.	Organism	Reads	RPM
	Enterobacter	3	0.24
	Enterobacter cloacae complex	3	0.24
	Enterobacter cloacae	1	0.08
	Serratia	3	0.24
	Serratia marcescens	2	0.16
	Serratia rubidaea	1	0.08
	Xanthomonas	3	0.24
	Xanthomonas campestris	1	0.08
	Roseomonas	3	0.24
	Roseomonas mucosa	2	0.16
	Bordetella	3	0.24
	Bordetella flabilis	1	0.08
	Veillonella	3	0.24
	Veillonella parvula	2	0.16
	Veillonella dispar	1	0.08
	Cronobacter	2	0.16
	Cronobacter sakazakii	1	0.08
	Pantoea	2	0.16
	Pantoea dispersa	2	0.16
	Haemophilus	2	0.16
	Haemophilus parainfluenzae	1	0.08
	Agrobacterium	2	0.16
	Agrobacterium tumefaciens	2	0.16
	Pannonibacter	2	0.16
	Pannonibacter phragmitetus	2	0.16
	Comamonas	2	0.16
	Ralstonia	2	0.16
	Ralstonia insidiosa	1	0.08
	Neisseria	2	0.16
	Neisseria subflava	1	0.08
	Pseudopropionibacterium	2	0.16
	Pseudopropionibacterium propionicum	2	0.16
	Citrobacter	1	0.08
	Escherichia	1	0.08
	Escherichia coli	1	0.08
	Shigella	1	0.08
	Pectobacterium	1	0.08
	Paracoccus	1	0.08
	Delftia	1	0.08
	Delftia tsuruhatensis	1	0.08
	Lautropia	1	0.08
	Lautropia mirabilis	1	0.08
	Paraburkholderia	1	0.08
	Fusobacterium	1	0.08

Patient No.	Organism	Reads	RPM
	Fusobacterium nucleatum	1	0.08
	Parabacteroides	1	0.08
	Parabacteroides distasonis	1	0.08
	Dolosigranulum	1	0.08
	Dolosigranulum pigrum	1	0.08
	Finegoldia	1	0.08
	Finegoldia magna	1	0.08
	Dermabacter	1	0.08
	Kytococcus	1	0.08
	Kytococcus sedentarius	1	0.08
	Mycobacterium	1	0.08
	Mycobacterium avium complex (MAC)	1	0.08
	Mycobacterium chimaera	1	0.08
	Mycolicibacterium	1	0.08
	Mycolicibacterium aurum	1	0.08
	Aspergillus	792	64.46
	Aspergillus niger	115	9.36
	Aspergillus flavus	64	5.21
	Aspergillus nidulans	17	1.38
	Aspergillus glaucus	16	1.3
	Aspergillus oryzae	14	1.14
	Alternaria	248	20.18
	Alternaria alternata	148	12.05
	Alternaria arborescens	26	2.12
	Trichoderma	61	4.96
	Trichoderma harzianum	43	3.5
	Trichoderma asperellum	6	0.49
	Trichoderma reesei	2	0.16
	Trichoderma virens	1	0.08
	Talaromyces	45	3.66
	Talaromyces pinophilus	3	0.24
	Talaromyces stipitatus	1	0.08
	Talaromyces atroroseus	1	0.08
	Malassezia	25	2.03
	Malassezia restricta	18	1.46
	Malassezia slooffiae	5	0.41
	Malassezia globosa	2	0.16
	Yarrowia	13	1.06
	Yarrowia lipolytica	13	1.06
	Fusarium	8	0.65
	Fusarium verticillioides	5	0.41
	Candida	3	0.24
	Candida parapsilosis	3	0.24
	Penicillium	3	0.24

Patient No.	Organism	Reads	RPM
	Penicillium rubens	2	0.16
	Penicillium digitatum	1	0.08
	Wickerhamomyces	2	0.16
	Wickerhamomyces anomalus	2	0.16
	Pichia	2	0.16
	Pichia kudriavzevii	2	0.16
	Schizophyllum	2	0.16
	Schizophyllum commune	2	0.16
	Debaryomyces	1	0.08
	Debaryomyces hansenii	1	0.08
	Meyerozyma	1	0.08
	Meyerozyma guilliermondii	1	0.08
	Phaeoacremonium	1	0.08
	Phaeoacremonium minimum	1	0.08
12	Anaerococcus	288	10.12
	Anaerococcus prevotii	25	0.88
	Staphylococcus	113	3.97
	Staphylococcus caprae	103	3.62
	Staphylococcus epidermidis	3	0.11
	Staphylococcus aureus	1	0.04
	Peptoniphilus	102	3.58
	Peptoniphilus harei	101	3.55
	Burkholderia	3	0.11
	Burkholderia cepacia complex	3	0.11
	Burkholderia contaminans	3	0.11
	Ralstonia	3	0.11
	Ralstonia insidiosa	2	0.07
	Finegoldia	3	0.11
	Finegoldia magna	3	0.11
	Methylobacterium	1	0.04
	Aspergillus	6	0.21
	Aspergillus campestris	2	0.07
	Aspergillus niger	1	0.04
	Aspergillus versicolor	1	0.04
	Penicillium	1	0.04
	Penicillium rubens	1	0.04
13	Staphylococcus	37824	1004.61
	Staphylococcus aureus	37312	991.01
	Staphylococcus agnetis	1	0.03
	Ralstonia	9	0.24
	Ralstonia insidiosa	9	0.24
	Burkholderia	2	0.05
	Burkholderia cepacia complex	2	0.05
	Burkholderia contaminans	1	0.03

Patient No.	Organism	Reads	RPM
	Acinetobacter	1	0.03
	Acinetobacter johnsonii	1	0.03
	Pseudomonas	1	0.03
	Pseudomonas stutzeri	1	0.03
	Brevundimonas	1	0.03
	Brevundimonas vesicularis	1	0.03
	Kocuria	1	0.03
	Kocuria rosea	1	0.03
	Rhodococcus	1	0.03
	Cutibacterium	1	0.03
	Cutibacterium acnes	1	0.03
14	Prevotella	25447	746.01
	Prevotella buccalis	17916	525.23
	Prevotella timonensis	2344	68.72
	Prevotella corporis	589	17.27
	Prevotella disiens	569	16.68
	Prevotella bivia	272	7.97
	Prevotella intermedia	198	5.8
	Prevotella amnii	190	5.57
	Prevotella dentalis	141	4.13
	Prevotella scopos	117	3.43
	Prevotella pallens	109	3.2
	Prevotella jejuni	106	3.11
	Prevotella denticola	26	0.76
	Prevotella oris	24	0.7
	Prevotella nigrescens	21	0.62
	Prevotella oulorum	18	0.53
	Prevotella melaninogenica	15	0.44
	Prevotella bryantii	13	0.38
	Prevotella enoeca	6	0.18
	Prevotella stercorea	3	0.09
	Prevotella fusca	1	0.03
	Anaerococcus	15306	448.72
	Anaerococcus hydrogenalis	11521	337.75
	Anaerococcus prevotii	94	2.76
	Anaerococcus tetradius	72	2.11
	Peptoniphilus	10634	311.75
	Peptoniphilus harei	1624	47.61
	Peptoniphilus rhinitidis	221	6.48
	Peptoniphilus coxii	73	2.14
	Peptoniphilus asaccharolyticus	2	0.06
	Pseudomonas	6468	189.62
	Pseudomonas aeruginosa	5978	175.25
	Finegoldia	4354	127.64

Patient No.	Organism	Reads	RPM
	Finegoldia magna	4354	127.64
	Streptococcus	516	15.13
	Streptococcus constellatus	440	12.9
	Streptococcus pyogenes	29	0.85
	Streptococcus pseudoporcinus	14	0.41
	Streptococcus agalactiae	6	0.18
	Streptococcus dysgalactiae	6	0.18
	Streptococcus pneumoniae	3	0.09
	Streptococcus equi	3	0.09
	Streptococcus anginosus	2	0.06
	Streptococcus suis	1	0.03
	Streptococcus intermedius	1	0.03
	Dermabacter	474	13.9
	Dermabacter hominis	150	4.4
	Corynebacterium	53	1.55
	Corynebacterium striatum	49	1.44
	Corynebacterium simulans	3	0.09
	Porphyromonas	52	1.52
	Porphyromonas endodontalis	34	1
	Porphyromonas gingivalis	16	0.47
	Helcococcus	35	1.03
	Helcococcus kunzii	35	1.03
	Bacteroides	16	0.47
	Bacteroides fragilis	6	0.18
	Bacteroides heparinolyticus	4	0.12
	Bacteroides thetaiotaomicron	3	0.09
	Bacteroides zoogleoformans	3	0.09
	Ralstonia	13	0.38
	Ralstonia insidiosa	9	0.26
	Ralstonia pickettii	3	0.09
	Odoribacter	12	0.35
	Odoribacter splanchnicus	12	0.35
	Campylobacter	5	0.15
	Campylobacter ureolyticus	2	0.06
	Fastidiosipila	4	0.12
	Fastidiosipila sanguinis	4	0.12
	Fusobacterium	3	0.09
	Fusobacterium necrophorum	3	0.09
	Treponema	3	0.09
	Treponema phagedenis	3	0.09
	Parvimonas	3	0.09
	Parvimonas micra	3	0.09
	Kocuria	3	0.09
	Kocuria rosea	2	0.06

Patient No.	Organism	Reads	RPM
	Lactobacillus	2	0.06
	Lactobacillus iners	2	0.06
	Mogibacterium	1	0.03
	Mogibacterium pumilum	1	0.03
	Gardnerella	1	0.03
	Gardnerella vaginalis	1	0.03
	Nocardiopsis	1	0.03
	Nocardiopsis dassonvillei	1	0.03
	Trichophyton	285	8.36
	Trichophyton rubrum	255	7.48
	Aspergillus	1	0.03
	Trichomonas	20	0.59
	Trichomonas vaginalis	20	0.59
15	Ralstonia	6	0.19
	Ralstonia insidiosa	4	0.13
	Ralstonia pickettii	1	0.03
	Acinetobacter	2	0.06
	Acinetobacter lwoffii	1	0.03
	Burkholderia	1	0.03
	Burkholderia cepacia complex	1	0.03
	Burkholderia contaminans	1	0.03
	Streptococcus	1	0.03
	Enterococcus	1	0.03
	Enterococcus cecorum	1	0.03
	Nocardiopsis	1	0.03
	Nocardiopsis dassonvillei	1	0.03
	Aspergillus	1	0.03
	Yarrowia	2	0.06
	Yarrowia lipolytica	2	0.06
16	Staphylococcus	110286	3285.96
	Staphylococcus caprae	103087	3071.47
	Staphylococcus epidermidis	683	20.35
	Staphylococcus aureus	309	9.21
	Staphylococcus capitis	134	3.99
	Staphylococcus warneri	86	2.56
	Staphylococcus gallinarum	54	1.61
	Staphylococcus haemolyticus	51	1.52
	Staphylococcus simulans	46	1.37
	Staphylococcus intermedius	31	0.92
	Staphylococcus hominis	22	0.66
	Staphylococcus lugdunensis	17	0.51
	Staphylococcus cohnii	16	0.48
	Staphylococcus xylosus	9	0.27
	Staphylococcus saprophyticus	7	0.21

Patient No.	Organism	Reads	RPM
	Staphylococcus equorum	1	0.03
	Staphylococcus pasteuri	1	0.03
	Corynebacterium	34988	1042.46
	Corynebacterium jeikeium	24395	726.85
	Corynebacterium resistens	1213	36.14
	Corynebacterium urealyticum	1037	30.9
	Corynebacterium segmentosum	248	7.39
	Corynebacterium striatum	157	4.68
	Corynebacterium falsenii	138	4.11
	Corynebacterium riegelii	137	4.08
	Corynebacterium macginleyi	116	3.46
	Corynebacterium simulans	110	3.28
	Corynebacterium aurimucosum	100	2.98
	Corynebacterium auriscanis	30	0.89
	Corynebacterium diphtheriae	26	0.77
	Corynebacterium accolens	26	0.77
	Corynebacterium propinquum	16	0.48
	Corynebacterium tuscaniense	14	0.42
	Corynebacterium halotolerans	7	0.21
	Corynebacterium camporealensis	6	0.18
	Corynebacterium ureicelerivorans	5	0.15
	Corynebacterium xerosis	2	0.06
	Corynebacterium humireducens	2	0.06
	Corynebacterium stationis	1	0.03
	Corynebacterium renale	1	0.03
	Kytococcus	18	0.54
	Kytococcus schroeteri	18	0.54
	Ralstonia	15	0.45
	Ralstonia insidiosa	9	0.27
	Ralstonia pickettii	3	0.09
	Dermabacter	15	0.45
	Dermabacter hominis	1	0.03
	Rhodococcus	13	0.39
	Rhodococcus erythropolis	4	0.12
	Trueperella	11	0.33
	Trueperella bernardiae	11	0.33
	Acinetobacter	7	0.21
	Acinetobacter johnsonii	5	0.15
	Acinetobacter gyllenbergii	1	0.03
	Brevundimonas	7	0.21
	Brevundimonas diminuta	1	0.03
	Brevundimonas vesicularis	1	0.03
	Streptomyces	5	0.15
	Pseudomonas	4	0.12

Patient No.	Organism	Reads	RPM
	Pseudomonas stutzeri	1	0.03
	Actinomyces	3	0.09
	Actinomyces viscosus	3	0.09
	Microbacterium	3	0.09
	Microbacterium oxydans	2	0.06
	Nocardiopsis	3	0.09
	Nocardiopsis dassonvillei	2	0.06
	Sphingomonas	2	0.06
	Sphingomonas koreensis	1	0.03
	Chromobacterium	2	0.06
	Moraxella	1	0.03
	Moraxella osloensis	1	0.03
	Burkholderia	1	0.03
	Burkholderia cepacia complex	1	0.03
	Burkholderia contaminans	1	0.03
	Veillonella	1	0.03
	Veillonella parvula	1	0.03
	Micrococcus	1	0.03
	Micrococcus luteus	1	0.03
	Aspergillus	14	0.42
	Aspergillus flavus	6	0.18
	Aspergillus niger	3	0.09
	Aspergillus glaucus	2	0.06
	Alternaria	4	0.12
	Alternaria alternata	3	0.09
	Yarrowia	2	0.06
	Yarrowia lipolytica	2	0.06
	Wickerhamomyces	1	0.03
	Wickerhamomyces anomalus	1	0.03
	Pichia	1	0.03
	Pichia kudriavzevii	1	0.03
	Trichoderma	1	0.03
	Trichoderma harzianum	1	0.03
	Malassezia	1	0.03
	Malassezia restricta	1	0.03
